# Loss of nuclear PTEN in HCV-infected human hepatocytes

**DOI:** 10.1186/1750-9378-9-23

**Published:** 2014-07-18

**Authors:** Wenjie Bao, Liliana Florea, Ningbin Wu, Zhao Wang, Krishna Banaudha, Jason Qian, Laurent Houzet, Rakesh Kumar, Ajit Kumar

**Affiliations:** 1 Department of Biochemistry and Molecular Medicine, The George Washington University School of Medicine, 2300 Eye Street, N.W., Washington, D.C. 20037, USA; 2 McKusick-Nathans Institute of Genetic Medicine, The Johns Hopkins University, Baltimore, MD, USA; 3 Molecular Virology Section, Laboratory of Molecular Microbiology, NIAID, NIH, Bethesda, MD, USA; 4 Current address: Metabolism Branch, NIH, Bethesda, MD, USA; 5 Current address: INSERM U1085-IRSET, Universite de Rennes1, Institut Federatif de Recherche 140, Rennes, France

**Keywords:** HCV infection, Nuclear PTEN restriction

## Abstract

**Background:**

Hepatitis C virus (HCV) infection is a major risk factor for chronic hepatitis and hepatocellular carcinoma (HCC); however, the mechanism of HCV-mediated hepatocarcinogenesis is not well understood. Insufficiency of PTEN tumor suppressor is associated with more aggressive cancers, including HCC. We asked whether viral non-coding RNA could initiate oncogenesis in HCV infected human hepatocytes. The results presented herein suggest that loss of nuclear PTEN in HCV-infected human hepatocytes results from depletion of Transportin-2, which is a direct target of viral non-coding RNA, vmr11.

**Methods:**

The intracellular distribution of PTEN in HCV-infected cells was monitored by immunostaining and Western blots of nuclear and cytoplasmic proteins. Effects of PTEN depletion were examined by comparing expression arrays of uninfected cells with either HCV-infected or vmr11-transfected cells. Target genes suggested by array analyses were validated by Western blot. The influence of nuclear PTEN deficiency on virus production was determined by quantitative analysis of HCV genomic RNA in culture media of infected hepatocytes.

**Results:**

Import of PTEN to the nucleus relies on the interaction of Transportin-2 and PTEN proteins; we show that depletion of Transportin-2 by HCV infection or by the introduction of vmr11 in uninfected cells results in reduced nuclear PTEN. In turn, nuclear PTEN insufficiency correlates with increased virus production and the induction of γ-H2AX, a marker of DNA double-strand breaks and genomic instability.

**Conclusion:**

An HCV-derived small non-coding RNA inhibits Transportin-2 and PTEN translocation to the nucleus, suggesting a direct viral role in hepatic oncogenesis.

## Background

*Phosphatase and tensin homologue deleted on chromosome 10* (PTEN) is one of the most commonly targeted tumor suppressor genes in human cancers, encoding a 403-residues dual specificity phosphatase with both lipid and protein phosphatase activities [1-5]. The lipid phosphatase activity of PTEN is a central negative regulator of the Phosphatidylinositol-3-kinase (PI3K) signal cascade for cell growth and proliferation. Deregulation of PTEN has been associated with a spectrum of metabolic disorders related to hepatocarcinogenesis [6]. A recent report [7] points to post-transcriptional silencing of PTEN by HCV core 3a protein as a possible mechanism of PTEN deregulation.

Nuclear insufficiency of PTEN has been shown to contribute to centromere destabilization and genomic instability [8,9], a hallmark of cancer, and nuclear PTEN depletion has been associated with more aggressive cancers [10-12]. In this study, we ask whether HCV infection initiates nuclear PTEN insufficiency and, in particular, whether viral non-coding RNA plays a part in regulating the intracellular redistribution of PTEN protein. We present evidence of inhibition of Transportin-2 by a viral non-coding RNA (vmr11). This inhibition restricts nuclear translocation of PTEN in HCV-infected human hepatocytes. We further show that restoring intracellular Transportin-2 levels rescues wild-type levels of nuclear PTEN. Based on the interaction between PTEN and Transportin-2, our results support a novel mechanism of regulation of nuclear PTEN translocation where down-regulation of Transportin-2 by vmr11 correlates with the nuclear exclusion of PTEN protein in HCV-infected human hepatocytes.

## Results

### Restriction of nuclear PTEN in HCV-infected cells

**Figure 1:**
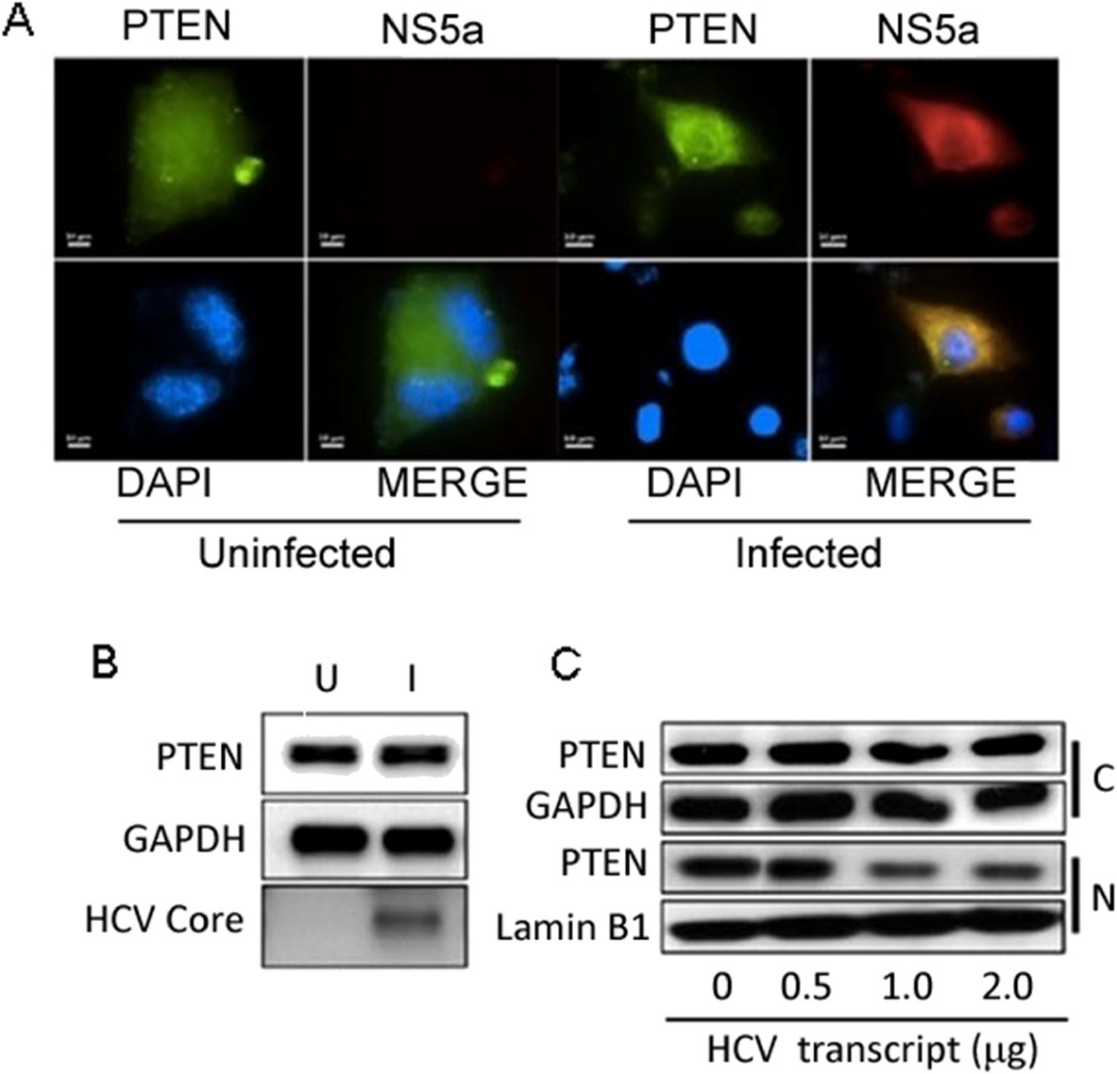
**HCV replication in human hepatocytes. (A)** Intracellular distribution of PTEN: Cells were visualized at day 3 post-transfection with HCV genomic RNA. The uninfected cells (left four panels) show uniform PTEN staining; HCV replication is marked with NS5A antigen (far right panel). Transfection efficiency of HCV genomic RNA was approximately 30%. **(B)** PTEN protein in HCV-infected cells: 1 × 10^5^ PPH cells were transfected with 1 μg H77 HCV1a genomic RNA, and the cells were collected at day five (U, uninfected; I, infected). Total cell protein was extracted using RIPA buffer; 20 μg of protein was analyzed on 10% precast Protein Gel; numbers underneath each lane indicate the relative values of PTEN. HCV replication is marked with viral core antigen (HCV Core). **(C)** Restriction of nuclear PTEN protein in HCV-infected cells: 1 × 10^5^ PPH cells were transfected with increasing amounts (0.5, 1.0, 2.0 μg) of input HCV1a genomic RNA, cells were harvested 5 days post-transfection and 10 μg each of nuclear or cytoplasmic protein were analyzed by Western blot. GAPDH and Lamin B1 were loading controls respectively, for the cytoplasmic or nuclear protein.

We examined the distribution of PTEN by immunofluorescence staining in human primary hepatocytes, before and after transfection with HCV1a genomic RNA [13]. Figure 1A (upper panels) shows a uniform distribution of PTEN in uninfected cells, whereas staining of nuclear PTEN was less uniformly distributed in HCV replicating cells. Virus replication in this assay was marked with immune staining for NS5A antigen (far right panel).To quantitatively analyze the intracellular distribution of PTEN protein, we performed Western blots of total cell protein, and of the nuclear and cytoplasmic fractions of HCV-infected cells (Figure 1B and 1C). There was no noticeable change in PTEN protein levels when we compared total cell proteins of HCV-replicating and control cells (Figure 1B; virus replication was marked by immunoblot for HCV core antigen). However, when we compared PTEN protein levels in the nuclear and the cytoplasmic fractions (Figure 1C), we observed marked inhibition of nuclear PTEN in HCV-replicating cells. These results suggest a progressive decline of nuclear PTEN in cells transfected with increasing amounts of viral genomic RNA (Figure 1C). The relatively unchanged levels of cytoplasmic PTEN and gradual decline in nuclear PTEN suggest, but do not prove, the deregulation of intracellular distribution of PTEN as possible mechanism of nuclear PTEN depletion in HCV-replicating cells. We therefore asked whether viral small non-coding RNAs might, directly or indirectly, modulate the intracellular distribution of the PTEN protein.

### HCV-derived small non-coding RNAs

**Figure 2:**
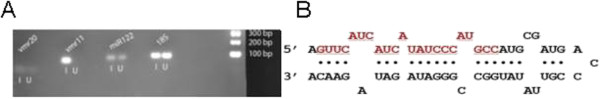
**The HCV derived viral small RNAs. (A)** Quantitation of vmrs: RT-PCR results show robust increase in vmr11 levels six days post-transfection with HCV genomic RNA, and only minimal change in vmr20. (I, infected; U, uninfected). As control, miR-122 level is minimally changed upon viral infection. (18S marker served as internal reference). **(B)** Structure of vmr11 RNA: Predicted secondary structure of vmr11 precursor. Mature vmr11 RNA is shown in red.

The positive sense HCV RNA genome is replicated in infected cells using the minus strand viral genomic RNA as a template. We reasoned that the conserved hairpin-loop structure of the minus strand genomic RNA, which is not engaged in translation, could serve as possible source of viral small non-coding RNAs (vmrs). To determine candidate vmrs, we employed bioinformatics methods to search the ~9,600 bp minus strand RNA genome for structurally conserved hairpins that could act as putative viral microRNA precursors. Sequence conservation at the ‘seed’ position in 37 GenBank HCV genomes as well as the configuration geometry of the hairpin arm and loop helped select 13 candidates, whose presence in HCV-infected cells were validated with qRT-PCR (see Methods and Additional file 1: Figure S1 (and Additional file 1: Table S1, for details). Here we discuss the functional validation of the 22 nt vmr11 RNA that was abundantly expressed in HCV infected human hepatocytes (Figure 2).

### Abundance of vmr11 varies with time post HCV infection

**Figure 3:**
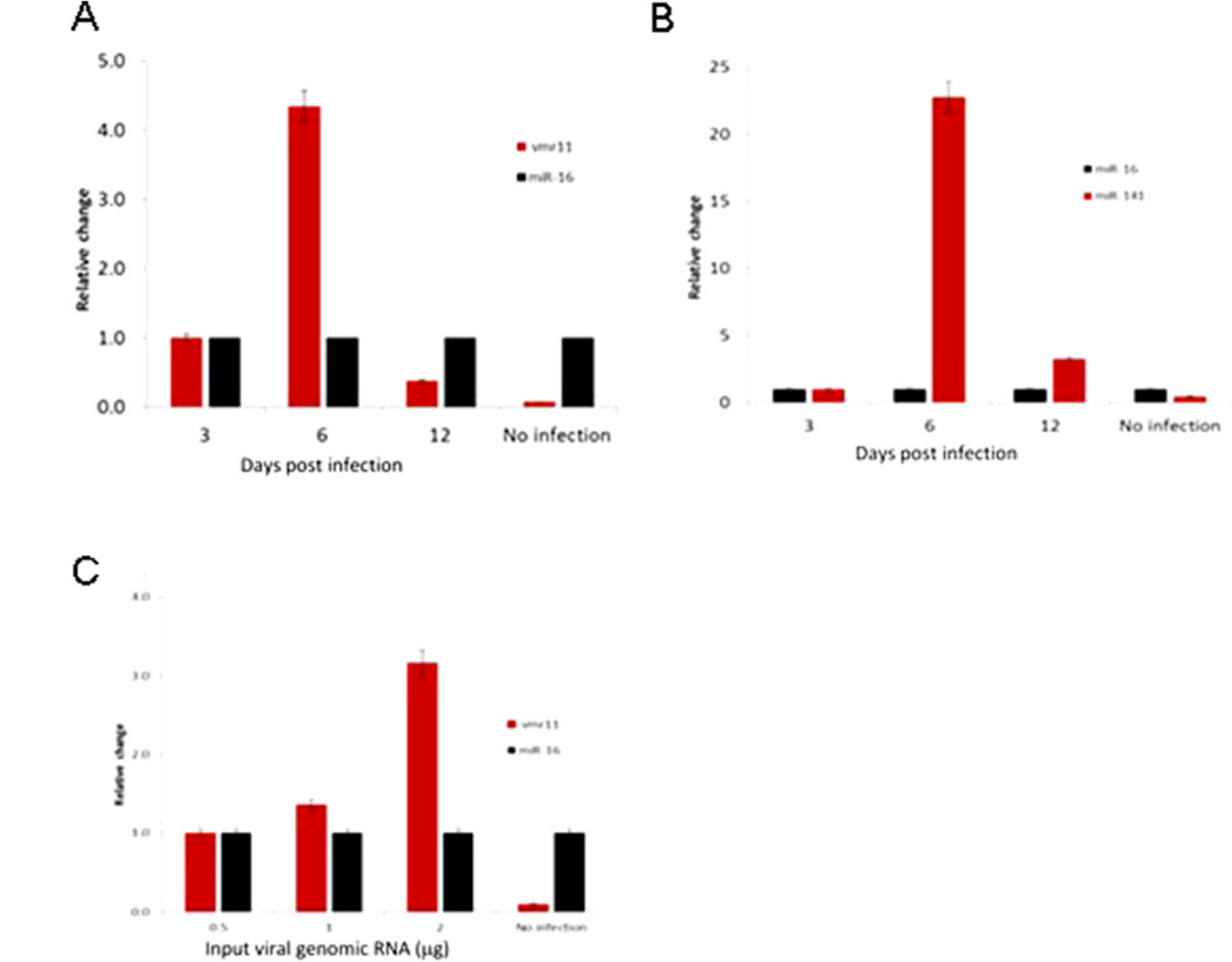
**Viral small non-coding RNA in HCV replicating cells. (A)** Expression of *vmr11 RNA***:** 1 × 10^5^ PPH cells were transfected with 1 μg of HCV1a genomic RNA and harvested on days 3, 6 and 12 post-transfection; vmr11 was monitored by qRT-PCR (normalized to miR-16, which does not change with viral infection). **(B)** MicroRNA-141 expression in cells transfected with HCV genomic RNA: qRT-PCR analysis of miR-141 in PPH cells transfected with increasing amounts of viral genomic RNA. **(C)** vmr11 expression with increasing HCV genomic RNA: qRT-PCR of vmr11 RNA in PPH cells transfected with increasing amounts of input viral genomic RNA (0.5, 1 and 2 μg) was analyzed five days post-transfection.

We reasoned that the HCV-derived vmr11 would be expected to accumulate with increasing duration of virus replication and increased input of viral genomic RNA. To test the prediction, we performed qRT-PCR analyses of vmr11 RNA in human hepatocytes (transfected with HCV genomic RNA) at 3, 6 and 12 days post-transfection (Figure 3A, B) and repeated the analyses in cells transfected with increasing amounts of HCV genomic RNA (Figure 3C). We observed the highest accumulation of vmr11 at day six post-transfection of human hepatocytes (Figure 3A). As reported earlier [13] HCV replication can be maintained for weeks with serial passages through naïve hepatocyte cultures. However, viral RNA replication in continuous cultures declines after a peak at six days, as does the HCV genomic RNA derived vmr11 (Figure 3A). The accumulation of vmr11 RNA also correlated with increasing amounts of viral genomic RNA transfected into human hepatocytes (Figure 3C). Serving as a control, we observed a parallel increase in host cell miRNA-141 (Figure 3B). It was previously reported that HCV replication correlates with miR-141-mediated post-transcriptional silencing of the DLC-1 tumor suppressor [14]. Conversely, expression of miR-16, which is not sensitive to HCV replication, remained unchanged in HCV-infected cells. While the mechanism behind the processing of the 22 nt vmr11 sequence is not completely understood, the increased production of vmr11 in parallel with viral replication indicates that vmr11 is derived from the HCV genomic RNA.

### Expression arrays of human hepatocytes transfected with vmr11 “mimic” oligonucleotides as compared to HCV-infected cells

To identify vmr11 target genes that may be involved in limiting nuclear PTEN translocation, we contrasted changes in gene expression in HCV-infected hepatocytes (shown as H77-1, -2 and -3 in three independent infections), and separately in cells transfected with vmr11 “mimic” oligonucleotides (mimic-1, -2 and -3), against uninfected (control) cells, using the *Agilent* microarray platform (Additional file 2). For each comparison, differential expression analyses revealed several hundred up- and down-regulated genes that could serve as potential markers for HCV infection-associated liver disease. Notably, the qualitative and quantitative changes in gene expression of cells transfected with vmr11 “mimic” oligonucleotides (vmr11 ‘mimic’ is defined as a synthetic 22nt oligomer identical in sequence with the ‘wild type’ vmr11) were similar to those in HCV-infected cells (Additional file 1: Figure S2).

Functional analyses using the KEGG database [15] revealed several pathways enriched among differentially expressed genes and in common between the two sets of comparisons (H77 and ‘mimic’). In particular, genes involved in lipid biosynthesis, intracellular signaling, and control of cell growth and patterning were enriched among the up-regulated genes; whereas genes involved in the regulation of cell motility, and members of the peroxisome proliferator activated receptors (PPAR) signaling pathway involved in lipid metabolism, were over-represented among the down-regulated genes (Additional file 3).

### Import of nuclear PTEN is sensitive to intracellular Transportin-2 levels

**Figure 4:**
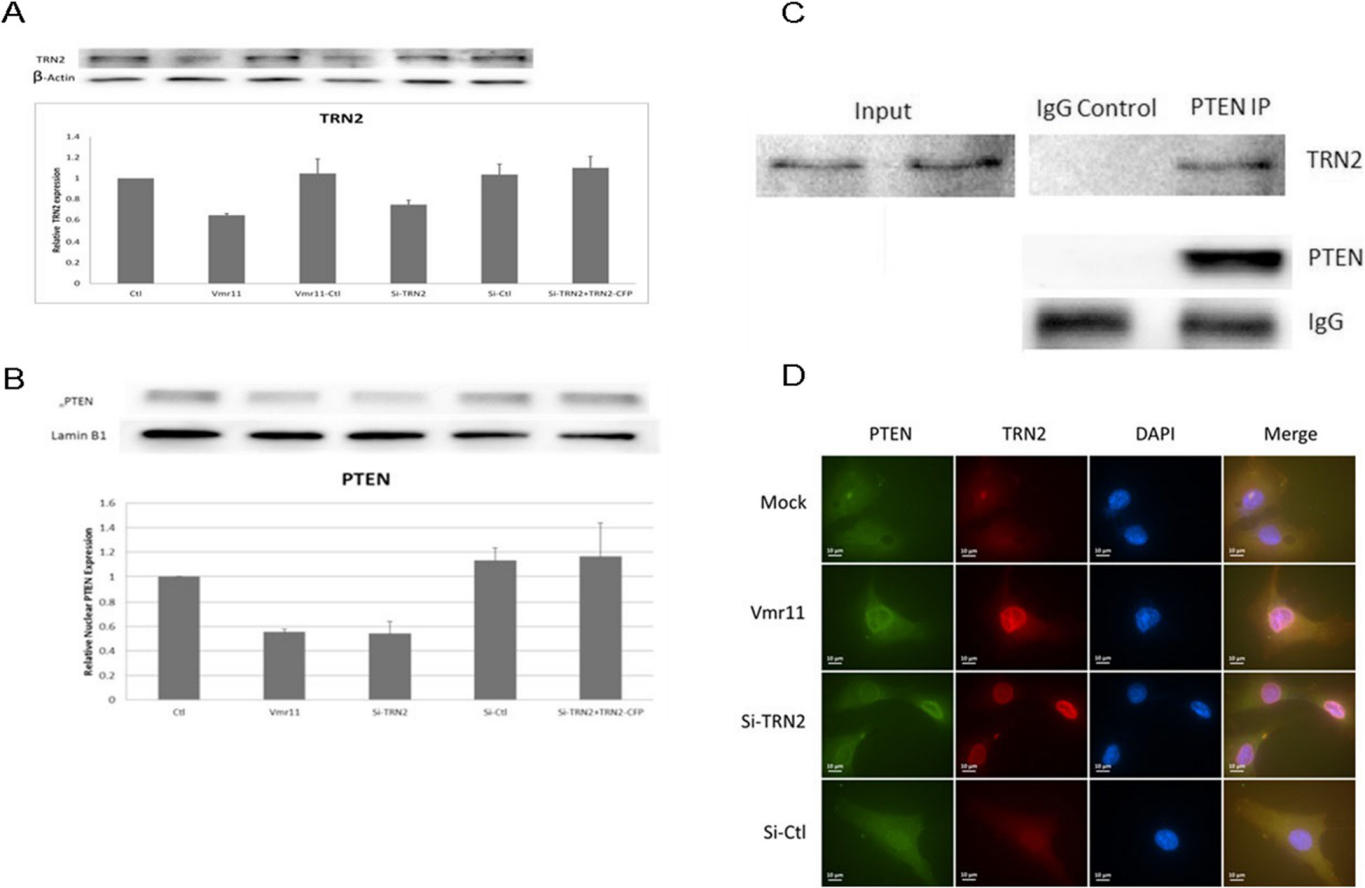
**Transportin-2 is required for PTEN translocation to the nucleus. (A)** Transportin-2 protein is inhibited by vmr11: Uninfected PPH cultures were transfected with 50 nM oligonucleotides (from left to right): scrambled vmr11 (Ctl), vmr11 mimic (vmr11), vmr11 mimic plus vmr11-antagomir (vmr11-Ctl), TRN-2 siRNA (Si-TRN-2), scrambled siTRN-2 (Si-Ctl) and siTRN-2 plus TRN-2 cDNA expression vector (Si-TRN-2 + TRN2-CFP). Western blots of TRN-2 protein from cells harvested 48 hours post-transfection were quantitated from three independent assays using β-Actin as loading control. **(B)** Nuclear PTEN is restricted by vmr11 or siTRN-2 knock-down: PPH cultures were transfected with 50 nM oligonucleotides (from left to right): scrambled vmr11 (ctrl), vmr11 “mimic” (vmr11), si-TRN2, scrambled si-TRN2 (si-ctl) or 1 μg TRN-2 cDNA expression vector following TRN-2 knock-down (si-TRN2 + TRN2-CFP). Transfections were repeated twice at 24 hour intervals and cells were harvested at 48 hour post-transfection; Western blot of nuclear PTEN was quantitated (with Lamin B1 as loading control); results shown are from three independent assays. **(C)** Transportin-2-PTEN protein interaction: Immunoprecipitates with PTEN antibody or IgG controls were analyzed by Western blot for PTEN or TRN-2 protein. PTEN-TRN-2 protein interaction is shown in the far right lane. **(D)** Suboptimal *PTEN-Transportin-2 protein complex is restricted at nuclear membrane*: Human primary hepatocytes were transfected with vmr11, Si-TRN2 and Si-Ctl oligonucleotides (50 nM of vmr11 mimic, twice at 0 hour and 24 hour). Cells were processed at 48 hrs post-transfection. PTEN is stained with FITC and TRN-2 with Texas Red.

Although the array results do not directly identify the vmr11-modulated genes, target analysis of 3’ UTRs of differentially down-regulated genes using the PITA prediction program [16] identified several potential vmr11 targets (Additional file 1: Table S2), including Transportin-2 (TNPO2, also known as Karyopherin β − 2b). Transportin-2 is known to interact with nucleocytoplasmic shuttling proteins and to regulate their nuclear import [17-20].Since both the results of expression arrays and the vmr11 targets predicted by PITA analysis of 3′ UTRs suggested that vmr11 could target Transportin-2 (TRN-2) expression, we verified TRN-2 protein levels in cells transfected with vmr11 oligonucleotides (Figure 4). We confirmed that TRN-2 was inhibited in human hepatocytes when the intracellular levels of vmr11 “mimic” oligonucleotides were artificially increased by transfection (Figure 4A, second lane from left). Countering the effects of vmr11 with the antisense oligonucleotides (vmr11-Ctl, third lane) restored Transportin-2 protein levels. As controls, we observed the inhibition of TRN-2 protein by siRNA knockdown (Si-TRN2, fourth lane from left), which was not seen in cells treated with scrambled siRNA (Si-Ctl, fifth lane from left). Restoring TRN-2 by transfecting Transportin-2 cDNA following siRNA knock down (Si-TRN-2 + TRN-2 CFP, far right lane) rescued intracellular Transportin-2 protein. Thus, introducing vmr11 oligonucleotides in uninfected cells appears to be sufficient to down-regulate Transportin-2 protein levels.Next, we asked whether the down-regulation of TRN-2 influences PTEN protein levels in the nucleus (Figure 4B). Western blots of nuclear PTEN show that knock-down of TRN-2 with siRNA (Si-TRN-2, third lane from left) is as effective in limiting nuclear PTEN as vmr11 ‘mimic’ oligonucleotides alone (second lane from left). As a control, scrambled siRNA (Si-Ctl, fourth lane from left) had no effect on nuclear PTEN restriction. Interestingly, restoring the intracellular TRN-2 by introducing Transportin-2 cDNA, following siRNA knock down of TRN-2 (Si-TRN-2 + TRN-2 CFP, far right lane), restored nuclear PTEN protein levels. These results indicate that the import of nuclear PTEN depends on Transportin-2.To determine if the direct TRN-2-PTEN interaction is at least partially responsible for the nuclear import of PTEN, we performed immunoprecipitation assays. As shown in Figure 4C, the PTEN antibody pull-down fraction (but not IgG controls) showed TRN-2-PTEN interaction. These results suggest that the nuclear translocation of PTEN depends on its binding to Transportin-2.To further explore the mechanism of restriction of nuclear PTEN in TRN-2 deficient cells, we examined the intracellular distribution of PTEN in cells either transfected with vmr11 oligonucleotides or in which TRN-2 was depleted by transfection of siRNA. As a control, immunofluorescent staining of mock- transfected cells and cells transfected with scrambled siRNA (si-Ctl) showed a uniform intracellular distribution of both PTEN (green FITC stain) and TRN-2 (Texas red). Interestingly, in TRN-2-deficient cells (either due to vmr11, or by si-TRN-2 knock-down), the PTEN-TRN-2 complex appears to be restricted to the nuclear membrane area (Figure 4D), suggesting that the nuclear translocation process is restrictive to suboptimal PTEN-TRN-2 protein complex.

### Functional domains of the HCV-derived vmr11

**Figure 5:**
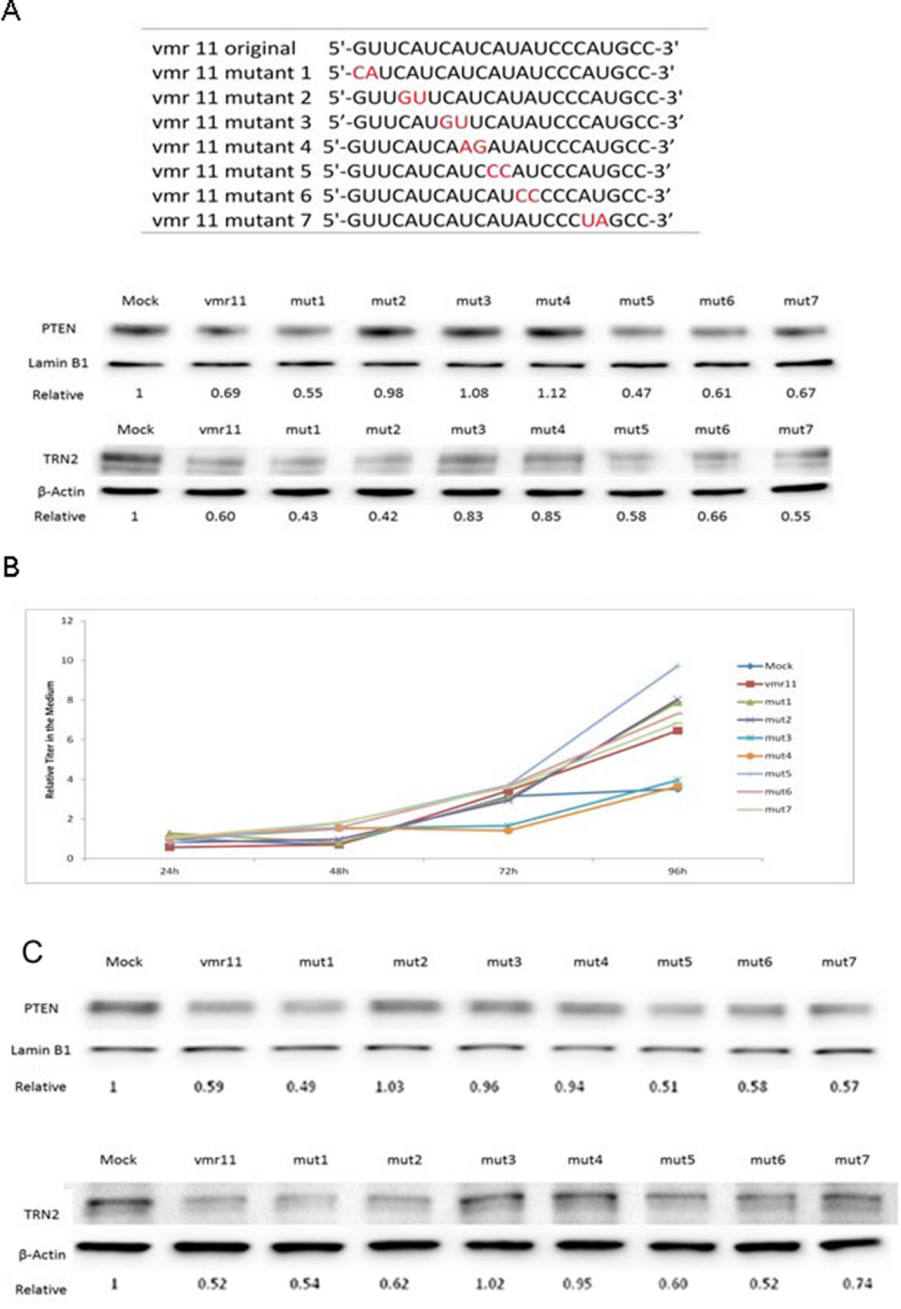
**Domains of vmr11 RNA required for PTEN or TRN-2 inhibition. (A)** vmr11mutants (upper panel): Base substitutions are shown in red. Western blots of PTEN and TRN-2 proteins (shown in lower panel): PPH cultures were transfected (twice, at 0 and 24 hours) with 50 nM vmr11 ‘mimic’ or one of the seven vmr11 mutants as indicated; total cell protein (for TRN-2), or the nuclear protein (for PTEN) was analyzed by Western blots. TRN-2 or PTEN protein levels were determined from two independent experiments, shown below each lane. β-actin or Lamin B1 was used as loading controls for TRN-2 and PTEN, respectively. **(B)** Virus production is positively correlated with loss of nuclear PTEN: 10^5^ PPH cells each were transfected with 1 μg HCV genomic RNA. A day later the cultures were either mock-transfected or transfected with vmr11 ‘mimic’ or with one of the seven vmr11 mutants (indicated in panel **A**). The oligonucleotide transfections were repeated twice (at 24 and 48 hour). Virus released in the culture media was harvested at 24-hour intervals as indicated. Viral RNA was analyzed by qRT-PCR. All viral RNA levels shown were normalized to the control, ‘mock transfected’ cells at 24 hour. **(C)** Loss of Nuclear PTEN and TRN-2 protein levels in cells transfected with vmr11 or vmr11 mutants: PTEN and TRN-2 proteins from the same cultures where virus released into the culture medium was quantitated **(panel B)**, were analyzed by Western blots. Relative values of nuclear PTEN or TRN-2 proteins are shown below each lane.

To determine ‘functional domains’ of vmr11 required for interaction with its TRN-2 target site, we examined base substitution mutants of vmr11 (Figure 5A, upper panel) and compared the effects on TRN-2 silencing and consequent restriction of nuclear PTEN by Western blot (Figure 5A, lower panel). In contrast with miRNA target recognition [21], the extreme 5′-sequence of vmr11 appears to be less critical for its TRN-2-target recognition. Indeed, inhibition of TRN-2 by vmr11 mutants 1 and 2 was similar to the wild type vmr11. Substitutions of nucleotides 7 and 8 (mutant 3) or nucleotides 9 and 10 (mutant 4), on the other hand, showed minimal inhibition of TRN-2, suggesting that nucleotides 7-10 of vmr11 contribute to the recognition of the TRN-2 target site. For the most part, nuclear PTEN protein levels also declined in parallel with the vmr11-targeted inhibition of TRN-2. As an exception, in cells transfected with mutant 2 (vmr11 base substitution from C_4_A_5_ to G_4_U_5_) the depletion of nuclear PTEN does not correlate with inhibition of Transportin-2. This finding suggests that mechanisms other than the TRN-2-PTEN interaction may contribute to restricting the nuclear PTEN translocation.

### Replication of HCV is positively correlated with restriction of nuclear PTEN

Experiments described thus far utilized vmr11 or vmr11 mutant oligonucleotides to artificially increase intracellular concentrations of vmr11. Next, we asked whether competition of vmr11 RNA produced *in vivo* in HCV-replicating cells would influence TRN-2 protein levels and limit nuclear PTEN. Significantly, such assays allowed us to address whether virus production (HCV genomic RNA recovered from the culture medium of infected cells) increased in response to limiting nuclear PTEN protein.

To compete vmr11 RNA *in vivo,* we transfected the various vmr11 mutants (shown in Figure 5A) into HCV-infected human hepatocytes. As shown (Figure 5B), base substitutions at nucleotides 7-10 (vmr11 mutants 3 and 4) that lost the capacity to recognize the TRN-2 target site, and hence showed no inhibition of TRN-2 protein or nuclear PTEN, minimally influenced HCV production. By contrast, vmr11 mutants (1, 2, 5, 6 and 7) that apparently do not interfere with TRN-2-target recognition, and hence allow the inhibition of TRN-2 and nuclear PTEN, favored virus production. These results suggest that depletion of nuclear PTEN favors virus production. Western blots of nuclear PTEN and TRN-2 proteins from the same cells that were scored for virus production are shown in Figure 5C. Overall, the results suggest that reduced levels of nuclear PTEN protein in HCV infected cells favors virus production; and that PTEN can be restored *in vivo* by the introduction of competing vmr11 mutant oligonucleotides.

### Depletion of nuclear PTEN correlates with increased γ − H2AX

**Figure 6:**
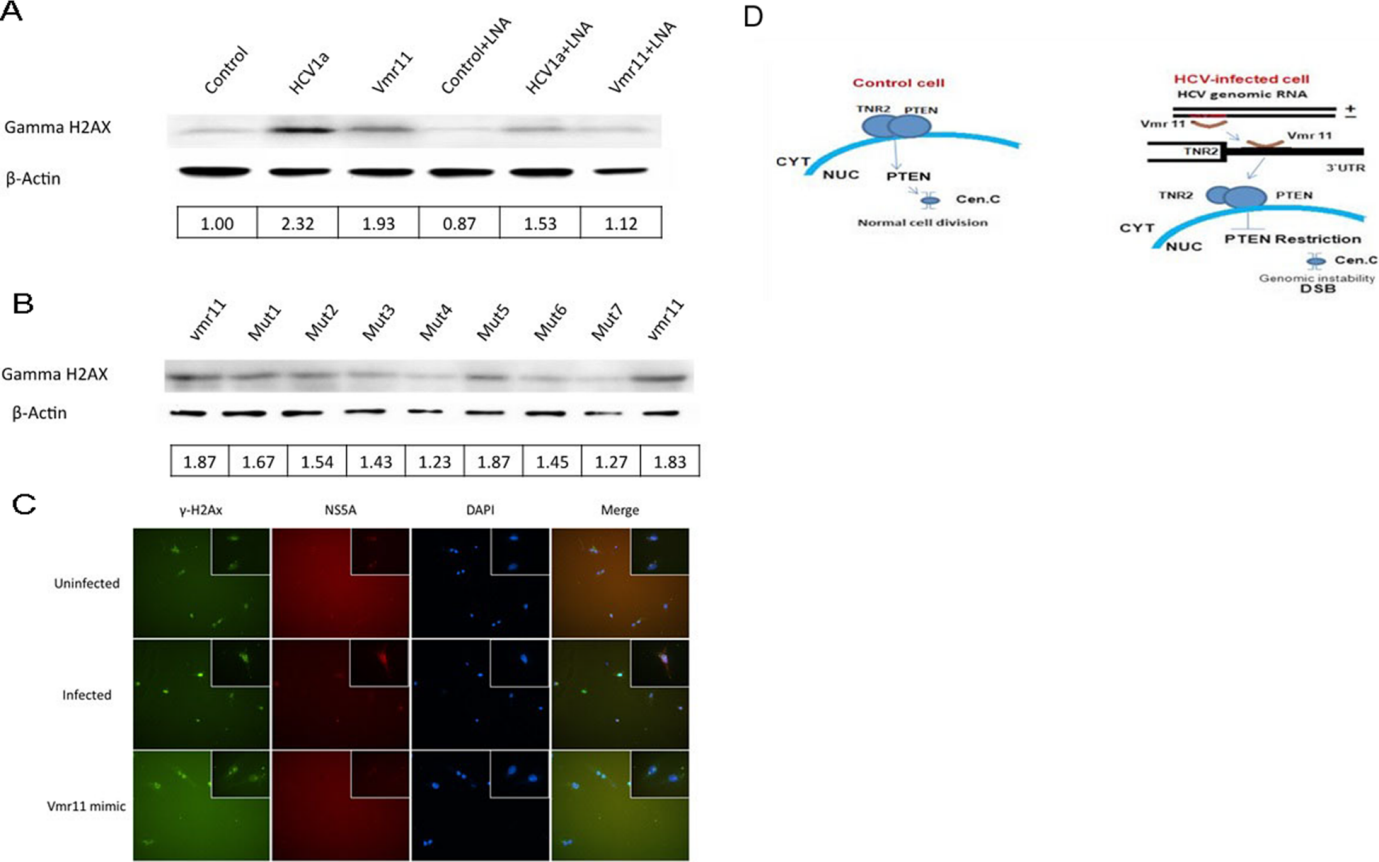
**Induction of γ-H2AX in HCV replicating or vmr11 transfected cells. (A)** γ-H2AX is induced by vmr11 in HCV infected cells: (from left to right), Human hepatocyte cultures were either mock transfected (Control), or transfected with 1 μg HCV1a (H77) genomic RNA, or with 50 nM vmiR11 “mimic” oligonucleotides. As controls, HCV1a genomic RNA or vmr11-transfected cultures were treated with 50 nM vmr11 antagomir (“LNA”). The cells were harvested 3 days post-transfection and analyzed by Western blot for γ-H2AX. The numbers beneath indicate relative values of γ-H2AX normalized to β-Actin loading control. **(B)** Induction of γ-H2AX correlates with vmr11 mutants that inhibit nuclear PTEN protein: Western blots (of cells transfected with vmr11 oligonucleotides, similar to the results shown in panel **A**) were compared with changes in γ-H2AX levels in cells transfected with either the WT vmr11, or with one of the vmr11 mutants (shown in Figure 5A). The numbers underneath represent relative values of γ-H2AX protein normalized to β-Actin loading control **(C)** Immunofluorescence staining of γ-H2AX (FITC) in HCV replicating or vmr11 transfected cells: Human hepatocytes were either transfected with HCV1a (1 μg viral genomic RNA; HCV replication is marked with NS5A viral antigen (Texas red stain), or transfected with vmr11 (50 nM) oligonucleotides, and processed for immunofluorescence viewing after 48 hours. γ-H2AX was stained with FITC and NS5A with Texas Red. Stained cell foci shown are at magnification 20x and 100x (inset). **(D)** Mechanism of regulation of nuclear PTEN deficiency by HCV: Schematic of nuclear PTEN restriction in HCV-infected cells that is mediated by translational silencing of Transportin-2 with vmr11.

Nuclear insufficiency of PTEN has previously been shown to destabilize the centromere complex and to lead to DNA double strand brakes (DSB) [8]. Accordingly, we asked if the loss of nuclear PTEN in HCV infected cells correlated with DSB, a hallmark of genomic instability. We assessed the level of DSBs by utilizing antibodies to the phosphorylated form of histone H2AX (γ-H2AX), a commonly used marker for DSBs [22,23]. Western blots of HCV-infected hepatocytes showed increased γ-H2AX levels as compared to uninfected hepatocytes (Figure 6A, second lane from the left). Consistent with the results shown in Figure 4B, introducing vmr11 “mimic” oligonucleotide in uninfected cell was sufficient for the induction of γ-H2AX (Figure 6A, third lane from the left). To validate that the vmr11-mediated inhibition of nuclear PTEN is in part responsible for inducing γ-H2AX, we countered the effects of vmr11 *in vivo* by introducing antisense vmr11 oligonucleotides in HCV-infected human hepatocytes and in the control cells transfected with vmr11 ‘mimic’ oligonucleotides. The results shown in Figure 6A suggest that the induction of γ-H2AX in PTEN-deficient cells is due to the intracellular vmr11 RNA.Analysis of vmr11 base-substitution mutants (Figure 5A) allowed the functional validation of vmr11 domains that are required for the inhibition of nuclear PTEN. It was of interest therefore, to test whether introducing vmr11 mutants would also induce changes in γ-H2AX levels in uninfected cells. As illustrated in Figure 6B base-substitution mutants of vmr11 (mutants 2, 3 and 4) that are less likely to inhibit nuclear PTEN protein levels do not induce γ-H2AX to the same extent as the wild type vmr11. Overall, the results implicate PTEN nuclear insufficiency to the induction of γ-H2AX.

In an attempt to correlate the effects of HCV infection or transfection with vmr11 RNA to γ-H2AX induction *in vivo,* we compared immunostaining of γ-H2AX in human hepatocytes either infected with HCV or transfected with vmr11 ‘mimic’ oligonucleotides. Immunostaining for NS5A viral antigen identified the HCV-infected cells (Figure 6C). Visual inspection of the HCV-infected cells and control cells that were transfected with vmr11 ‘mimic’ oligonucleotides identified relatively enhanced γ-H2AX staining nuclei. Overall, these results are consistent with the interpretation that depletion of nuclear PTEN in human hepatocytes, either due to HCV infection or by artificially increasing intracellular vmr11 RNA in uninfected cells, results in the induction of γ-H2AX.To conclude, inhibition of Transportin-2 by the viral non-coding RNA, vmr11 limits PTEN translocation to the nucleus and is sufficient to induce γ-H2AX an indicator of DSB and genomic instability in HCV-infected human hepatocytes (an overview of the results, is depicted in Figure 6D).

## Discussion

One of the critical steps in virus life cycle is their ability to modulate host defenses that favor virus propagation. For oncogenic virus such as HCV, a challenge is to define direct viral role in the depletion of PTEN tumor suppressor that correlates with more aggressive cancer, including HCC. The results described here suggest a novel mechanism of regulation of intracellular distribution of PTEN protein by viral non-coding RNA directed suppression of Transposrtin-2. Viral non-coding RNA mediated inhibition of Transportin-2 and the loss of nuclear PTEN in HCV-infected human hepatocytes implicate a direct viral role in oncogenic transformation. Consistent with that interpretation is our observation that HCV mediated nuclear insufficiency of PTEN correlates with DSB and genomic instability and efficient virus production.

Small RNA-based mechanisms, such as RNA interference (RNAi), play important roles in regulating the course of viral infection and viral pathogenesis in plants and animals [24-29]. Examples of virus-derived microRNAs have been reported in the human cytomegalovirus, Epstein Barr virus, Kaposi Sarcoma-associated virus, peach latent mosaic viroid [30,31], as well as in two viruses with RNA genomes, HIV-1 and BLV [27,32]. An earlier study [33] employing high-throughput sequencing predicted, albeit did not functionally validate, the presence of small viral RNAs derived from both positive and negative sense genomic RNA of several viruses, including HCV. By analyzing the conserved hairpin-loop structures of the HCV minus strand genomic RNA as a source of viral microRNA, we identified novel viral small non-coding RNA, vmr11, and have functionally validated the vmr11-dependent restriction of nuclear PTEN.

PTEN is a haploinsufficient tumor suppressor; partial depletion of PTEN is expected to profoundly influence cell cycle deregulation and genomic instability, hallmarks of oncogenic susceptibility. A number of mechanisms have been proposed to explain nucleocytoplasmic partitioning of PTEN [34-36]; however, there are no examples of PTEN restriction mediated by a human oncogenic virus. PTEN lacks both the conventional nuclear import (nuclear localization signal, NLS) and the nuclear export (NES) motifs [1,35]. No mutation has been reported among all the cases of nuclear PTEN ‘mis-localization’ examined [1], leaving open the possibility that nuclear translocation of PTEN may be regulated by ‘non-genetic’ mechanisms such as protein-protein interaction involving TRN-2.

PTEN deregulation plays a significant role in hepatopathogenesis [37,38]. The proposed role of HCV core 3a in modulating the formation of PTEN-dependent large lipid droplets in hepatocytes [7] implicates a direct viral role in liver disease. Adding to these studies, the results described herein support a novel mechanism for regulation of PTEN translocation to the nucleus in HCV-infected cells. Specifically, nuclear insufficiency of PTEN in HCV-infected cells results from the depletion of Transportin-2, which is mediated by a novel viral non-coding RNA, vmr11. Reduced levels of Transportin-2 in cells that are either transfected with vmr11 ‘mimic’ oligonucleotides, or suffer siRNA knock-down of endogenous TRN-2, restrict the Transportin-2 and PTEN complex at the nuclear membrane. Collectively, these findings point to a novel mechanism of regulation of intracellular distribution of PTEN that is mediated by Transportin-2, a direct target of vmr11. The observation that the TRN-2-PTEN protein complex is restricted at the nuclear membrane leaves open the possibility that factor(s) other than protein-protein interaction may be required for the PTEN translocation to the nucleus.

Perhaps the most intriguing consequence of nuclear PTEN insufficiency in HCV-infected cells is the induction of γH2AX, a major player in the recognition and repair of DNA double-strand breaks (DSB) and a hallmark of genomic instability and cancer susceptibility [8,39,40]. We observed enhanced staining foci of γH2AX-positive cells in HCV-infected or vmr11-transfected human hepatocytes. Whether DSB related to the nuclear PTEN insufficiency in HCV infection serve as an early indicator of HCV infection-associated hepatocellular carcinoma can be pursued in future studies in an animal model of HCV-infection associated HCC.

## Methods

### Post-attachment primary hepatocytes (PPH)

A sustained primary human hepatocytes culture system was recently described [13]. Briefly, a co-culture of Hepatic Stellate cell line (CFSC-8B) was used as a ‘feeder layer’, to provide the extracellular matrix for efficient attachment of human primary hepatocytes suspension. The co-culture was maintained in a serum-free Hepatocyte-Defined Medium (HDM) for 30 days, during which the stellate cells (that require serum for their replication) are largely depleted. The primary human hepatocytes form “spheroids” that can be dispersed (with 0.05% trypsin treatment) and propagated as monolayers (independent of stellate cells), as ‘Post-attachment Primary Hepatocytes’ (PPH) in HDM supplemented with 1% FBS. The PPH cultures are dispersed with 0.05% trypsin and reseeded at 1:3 dilutions at weekly intervals.

### Bioinformatics prediction of HCV small non-coding RNAs

The 9,646 bp negative sense HCV type 1 genome (GenBank accession: NC_004102) was divided, starting every 20 bp, into fixed size segments of length L = 70 (80, 90 and 100). RNAfold [41] was employed to test each short sequence to determine stable hairpin structures that can form viral small RNA precursors. Hairpins that scored in the top 5% of the score distributions (determined by comparison to simulated random samples) were analyzed for conserved secondary structure by comparing 37 HCV type 1 complete genome sequences. Lastly, putative viral microRNA sequences were determined starting from conserved sequences 7 bp or longer (‘seeds’) that could be extended to mature 20-25 viral small RNAs (see Additional file 1 for details).

### Transfection of PPH using H77 run-off transcript

pCV-H77c plasmid DNA was linearized for run-off transcription of full length genomic RNA using T7 Ribomax Express (Promega). 5×10^5^ PPH cells were transfected with 1 μg of the H77 transcript using the Fugene 6 (Promega) procedure. Virus replication was monitored by quantitation of genomic equivalents (GE) of HCV RNA or by immune blotting for NS5A or HCV core antigens.

### Real time Quantitative PCR of viral small RNAs

1 μg of total RNA was reverse-transcribed using QuantiMir RT Kit (System Biosciences). The cDNA was diluted (100-fold) and amplified using PCR Supermix (Invitrogen) or iTaq SYBR Green Supermix with Rox (Bio-Rad) on GeneAmp PCR System 9700 or on ABI 7300 Real Time PCR system.

### Subcellular protein fractionation and Western blot

PPH cells were lysed with either RIPA buffer supplemented with 1X protease inhibitor cocktail (Roche), or with NE-PER nuclear and cytoplasmic extraction reagents (Thermo Pierce) for fractionations of nuclear or cytoplasmic proteins. Normally, 10 μg of protein was analyzed on 10% precast Mini-PROTEIN gel (BIO-RAD). The gels were transferred to PVDF membrane, blocked with 5% non-fat milk and probed with antibodies against PTEN (ab#79156 1:1000), GAPDH (ab8245, 1:5000), Lamin-B1 (ab#16048, 1:500), HCV core (ab50288, 1:1000) or γ-H2AX (ab11174, 1:1000).

### Immunostaining

5×10^4^ of HCV-infected or uninfected PPH cells were seeded into six-well plates with sterilized cover glasses one day before staining. The cells were washed with PBS and fixed with 4% paraformaldehyde, washed and permeabilized with methanol at -20°C, blocked with 1% BSA and stained with primary antibody against PTEN (ab32199, 1:100) and NS5A (ab20342, 1:100); followed by FITC or Texas Red conjugated secondary antibodies.

### Quantitation of virus from the culture medium of infected cells

QIAamp Viral RNA Mini Kit (QIAGEN) was used to extract RNAs from 140 μl of the culture medium of HCV–infected (or control) cells. The cDNA was prepared using QuantiTect Rev Transcription Kit (QIAGEN) and was analyzed with primers that target the 5’ end of HCV (positive sense) genomic RNA. Primer sequences are H77-QRT-Forward (TGTGGAGCTGAGATCACTGG) and H77-QRT-Reverse (CCGCCTTATCTCCACGTATT).

### Statistical analysis

All Quantitative Real time PCR and densitometry data were analyzed using Microsoft Excel 2010.

### RNA labeling and array hybridization

Sample labeling and array hybridization were performed according to the Agilent One-Color Microarray-Based Gene Expression Analysis protocol (Agilent Technology).

### Data analysis

The Agilent Feature Extraction software (version 11.0.1.1) was used to analyze acquired array images. Quantile normalization and subsequent data processing were performed with the GeneSpring GX v12.0 software package (Agilent Technologies). Differentially expressed mRNAs were identified through Fold Change filtering (cutoff 2.0). Pathway analyses were performed using the standard enrichment computation method. Lastly, 3′UTRs of GenBank RefSeq genes for vmr11 putative target analysis with PITA were extracted from the UCSC Genome Browser tables. (Details are provided in Additional file 1).

## Competing interests

The authors declare that they have no competing interests.

## Authors’ contributions

WB, performed the experiments; LF, performed the bioinformatics; NW, performed the experiments; ZW, performed the experiments; KB, performed the cell culture experiments; JQ, performed the experiments; LH, performed the experiments; RK, contributed the sequencing experiments, AK, wrote the manuscript. All authors read and approved the final manuscript.

## Supplementary Material

Additional file 1 **Prediction of small non-coding RNAs from HCV genomic RNA. Figure S1.** Schematic representation for predicting conserved structural domains of HCV1a genomic RNA and the mature vmr sequences. **Table S1.** Candidate vmr sequences were derived from the HCV1a negative sense genomic RNA. **Figure S2.** Gene expression in human hepatocytes transfected with vmr11 ‘mimic’ are similar to those in HCV infected cells. (A) Down-regulated genes. (B) Up-regulated genes. **Table S2.** vmr11 targets in HCV infected (H77) and vmr11 transfected hepatocytes, predicted by PITA. **Figure S4.** vmr11binding sites on the TRN-2 3′UTR. Vmr11 sequence is shown in red, with the seed region in lowercase letters, PITA-predicted binding site is underlined. Click here for file

Additional file 2 Differentially expressed genes in HCV infected vmr11 RNA transfected human hepatocytes. Click here for file

Additional file 3 **Genes enriched in HCV infected and vmr11 RNA transfected human hepatocytes.** Inhibition of nuclear PTEN in HCV-infected human hepatocytes, by Bao *et al.* Click here for file
