# Supplementary material for: Loss of nuclear PTEN in HCV-infected human hepatocytes
Source: Infect Agent Cancer. 2014 Jul 18;9:23. doi: 10.1186/1750-9378-9-23 (PMC4114100; doi:10.1186/1750-9378-9-23)
Supplement: Additional file 1 — Prediction of small non-coding RNAs from HCV genomic RNA. Figure S1. Schematic representation for predicting conserved structural domains of HCV1a genomic RNA and the mature vmr sequences. Table S1. Candidate vmr sequences were derived from the HCV1a negative sense genomic RNA. Figure S2. Gene expression in human hepatocytes transfected with vmr11 ‘mimic’ are similar to those in HCV infected cells. (A) Down-regulated genes. (B) Up-regulated genes. Table S2. vmr11 targets in HCV infected (H77) and vmr11 transfected hepatocytes, predicted by PITA. Figure S4. vmr11binding sites on the TRN-2 3′UTR. Vmr11 sequence is shown in red, with the seed region in lowercase letters, PITA-predicted binding site is underlined. [file 1750-9378-9-23-S1.doc]

**ADDITIONAL FILE 1**

**Computational prediction of small non-coding RNAs from HCV genomic RNA**

To predict small viral RNA sequences from HCV genomic RNAs, we filtered the genomic sequences on the basis of the following criteria: 1) It must form a stable hairpin-loop structure; 2) The hairpin-loop structure must be conserved across HCV strains, possibly by compensatory mutations that preserve the base-pairings; and 3) The sequence of the mature *vmr* within the hairpin must also be conserved, to retain the ability to bind to its target. We downloaded from GenBank complete HCV1a genomic sequences and divided one of the sequences (NC_004102), deemed reference, into 70-100 bp sequence fragments with 20bp shift (overlap) (**Figure S1**). Each fragment was then analyzed using the program *RNAfold* to seek stable hairpin-loop structures that could be suitable substrates for processing small RNAs by host cell nucleases. All selected hairpins were cross-compared with the remaining HCV genomic sequences for conservation of the sequence and secondary structures. Eventually, thirteen candidate viral microRNAs (*vmr*) (**Table S1**) were selected for further study, some of which were validated with RT-PCR (**Figure S2**).


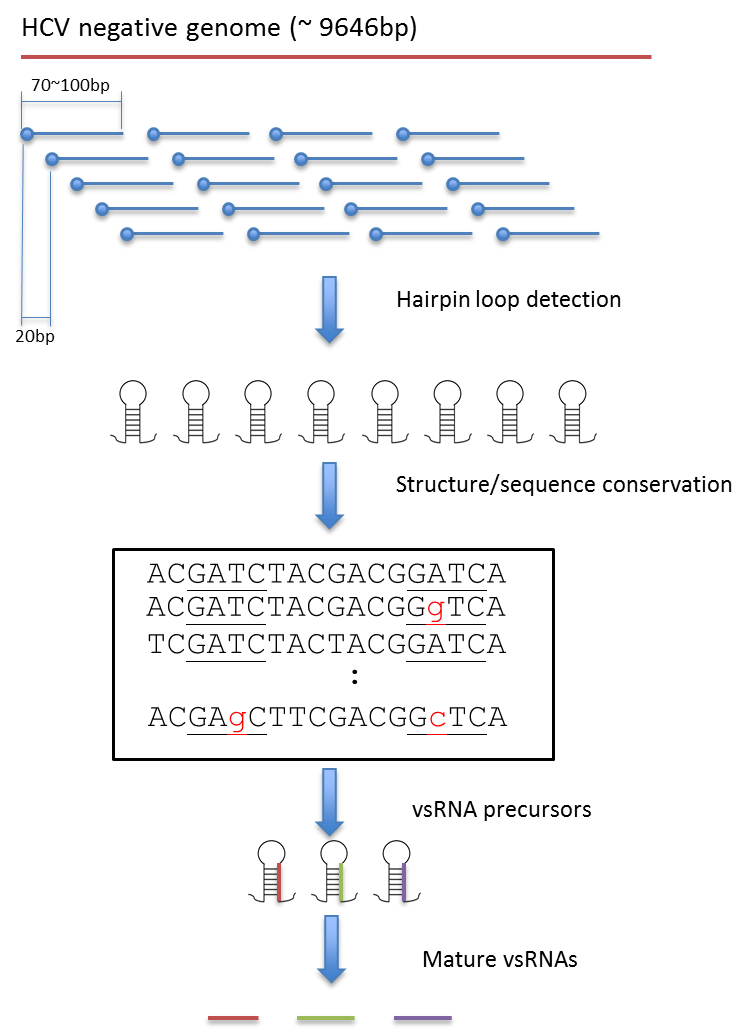


**Figure S1:** *Schematic representation of the bioinformatics approach for predicting conserved structural domains of HCV1a genomic RNA, precursors of viral small non-coding RNAs, and the mature vmr sequences.* Fixed-size (70, 80, 90 and 100 bp) segments of the negative strand of the HCV1a reference genome (NC_004102) were evaluated for their potential to encode viral microRNAs by first detecting segments that contain stable hairpins, then testing them for conservation of the hairpin structures in 37 HCV genotype 1a genomes. Lastly, sequence conservation and position within the precursor’s ‘arm’ was used to locate the ‘seed’ and the mature microRNA sequence.

**Table S1.** *Thirteen candidate vmr sequences were derived from the HCV1a negative sense genomic RNA.* The vmr sequences and their location (indicated on the 9,646 bp negative strand HCV genomic RNA) are shown. (**S**: Overall sequence conservation; **R**: Hairpin conservation; **RSS**: Combined structure and ‘seed’ sequence conservation.)

**Figure S2:** *Gene expression changes in human hepatocytes transfected with vmr11 ‘mimic’ are largely similar to those in HCV infected cells.* ***(A)*** Down-regulated genes. ***(B*)** Up-regulated genes.

262

↓H77: 710

↓mimic: 377

203

↑H77: 900

↑ mimic: 341

(A)

(B)

Table S2: *Putative vmr11 targets among down-regulated genes in HCV infected (H77) and vmr11 transfected (’mimic’) hepatocytes, predicted by PITA (score cutoff -10.0).* Sequences in common to the two comparisons (H77 and ‘mimic’) are shown in bold font.

| RefSeq Accession | PITA score | Function |
| --- | --- | --- |
| Genes down-regulated in H77: | | |
| NM_000457.4 | -19.30 | Hepatocyte nuclear factor 4, alpha (HNF4A), transcript variant 2, mRNA |
| **NM_002103.4** | -14.63 | Glycogen synthase 1 (muscle) (GYS1), transcript variant 1, mRNA |
| NM_145892.2 | -14.17 | Ataxin 2-binding protein 1 (A2BP1), transcript variant 2, mRNA |
| NM_006755.1 | -13.04 | Transaldolase 1 (TALDO1), mRNA |
| **NM_001099439.1** | -12.31 | EPH receptor A10 (EPHA10), transcript variant 3, mRNA |
| NM_025152.2 | -12.16 | Nucleotide binding protein-like (NUBPL), mRNA |
| **NM_205863.2** | -12.07 | Par-3 partitioning defective 3 homolog B (C. elegans) (PARD3B), transcript variant a, mRNA |
| **NM_013433.4** | -11.85 | Transportin 2 (TNPO2), transcript variant 2, mRNA |
| **NM_133458.2** | -11.34 | Zinc finger protein 90 homolog (mouse) (ZFP90), mRNA |
| NM_022336.3 | -10.96 | Ectodysplasin A receptor (EDAR), mRNA |
| NM_000728.3 | -10.76 | Calcitonin-related polypeptide beta (CALCB), mRNA |
| NM_006537.2 | -10.67 | Ubiquitin specific peptidase 3 (USP3), mRNA |
| **NM_020927.1** | -10.60 | Vesicle amine transport protein 1 homolog (T. californica)-like (VAT1L), mRNA |
| NM_001142523.1 | -10.47 | Interleukin-1 receptor-associated kinase 3 (IRAK3), transcript variant 2, mRNA |
| NM_000872.4 | -10.06 | 5-hydroxytryptamine (serotonin) receptor 7 (adenylate cyclase-coupled) (HTR7), transcript variant a, mRNA |
| **Genes down-regulated in ‘mimic’** | | |
| **NM_000457.3** | -19.30 | Hepatocyte nuclear factor 4, alpha (HNF4A), transcript variant 2, mRNA |
| **NM_002103.4** | -14.63 | Glycogen synthase 1 (muscle) (GYS1), transcript variant 1, mRNA |
| NM_002923.3 | -13.63 | Regulator of G-protein signaling 2, 24kDa (RGS2), mRNA |
| NM_005079.2 | -12.49 | Tumor protein D52 (TPD52), transcript variant 3, mRNA |
| NM_001002231.1 | -12.46 | Kallikrein-related peptidase 2 (KLK2), transcript variant 2, mRNA |
| **NM_001099439.1** | -12.31 | EPH receptor A10 (EPHA10), transcript variant 3, mRNA |
| **NM_205863.2** | -12.07 | Par-3 partitioning defective 3 homolog B (C. elegans) (PARD3B), transcript variant a, mRNA |
| **NM_013433.4** | -11.85 | Transportin 2 (TNPO2), transcript variant 2, mRNA |
| **NM_133458.2** | -11.34 | Zinc finger protein 90 homolog (mouse) (ZFP90), mRNA |
| NM_001039937.1 | -11.08 | Integrator complex subunit 6 (INTS6), transcript variant 2, mRNA |
| **NM_020927.1** | -10.60 | Vesicle amine transport protein 1 homolog (T. californica)-like (VAT1L), mRNA |

**Computational prediction of vmr11 binding sites on TRN-2 3’UTR**

Most microRNA target prediction programs use conservation of the putative target site in other species to narrow down candidates, but this requirement is not to be expected in our case, where the HCV infection may be restricted to human. In contrast, the program PITA [1] takes into account the thermodynamics of the microRNA-mRNA hybrid as well as the target site accessibility determined by base-pairs interactions within the mRNA to predict target sites, and therefore is particularly well suited for our application. Using PITA, we identified eight putative targets of vmr11 along the TRN-2 3’UTR, of which the strongest is located in the 3474-3466 region (**Figure S4**).

[1] Kersetz M, Iovino N, Unnerstall U, Gaul U, Segal E (2007) The role of site accessibility in microRNA target recognition. *Nature Genetics 39:1278—84.*


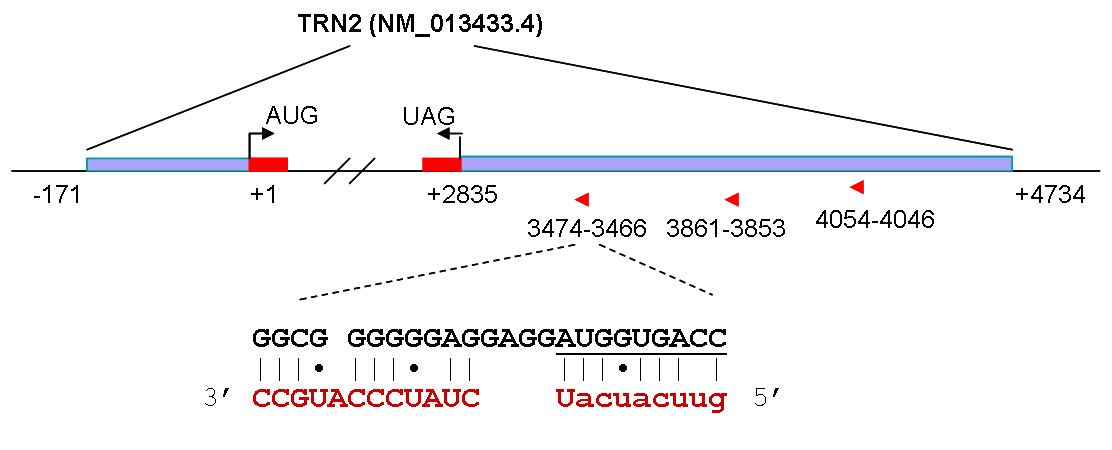


**Figure S4**. *Predicted vmr11 binding sites on the TRN-2 3’UTR.* PITA revealed eight possible binding sites, of which the strongest is located at 3474-3466 with the UTR. Vmr11 sequence is shown in red, with the seed region in lowercase letters, and the PITA-predicted binding site anchor is underlined.
