# Supplementary material for: Loss of nuclear PTEN in HCV-infected human hepatocytes
Source: Infect Agent Cancer. 2014 Jul 18;9:23. doi: 10.1186/1750-9378-9-23 (PMC4114100; doi:10.1186/1750-9378-9-23)
Supplement: Additional file 3 — Genes enriched in HCV infected and vmr11 RNA transfected human hepatocytes. Inhibition of nuclear PTEN in HCV-infected human hepatocytes, by Bao et al. [file 1750-9378-9-23-S3.docx]

### Additional file 3: Inhibition of nuclear PTEN in HCV-infected human hepatocytes, by Bao *et al.*

**Table S1.** KEGG pathways enriched among genes differentially expressed between uninfected (Control) and HCV-infected (H77) or vmiR11 mimic-transfected ('mimic') hepatocytes.

#### H77-vs-Control (down)

| **PathwayID** | **Definition** | **Fisher-Pvalue** | **SelectionCounts** | **SelectionSize** | **Count** | **Size** | **FDR** | **Enrichment_Score** | **Genes** |
| --- | --- | --- | --- | --- | --- | --- | --- | --- | --- |
| [hsa04141](http://www.genome.jp/dbget-bin/show_pathway?hsa04141+573+581+821+1649+51009+64215+30001+3320+22824+3312+10960+64374+6745+6747+201595+7322+7325+10277) | Protein processing in endoplasmic reticulum - Homo sapiens (human) | 1.493082e-004 | 18 | 256 | 166 | 6205 | 3.747637e-002 | 3.825916e+000 | [BAG1](http://www.genome.jp/dbget-bin/www_bfind_sub?mode=bfind&max_hit=10000&dbkey=hsa&keywords=BAG1&mode=bfind)//[BAX](http://www.genome.jp/dbget-bin/www_bfind_sub?mode=bfind&max_hit=10000&dbkey=hsa&keywords=BAX&mode=bfind)//[CANX](http://www.genome.jp/dbget-bin/www_bfind_sub?mode=bfind&max_hit=10000&dbkey=hsa&keywords=CANX&mode=bfind)//[DDIT3](http://www.genome.jp/dbget-bin/www_bfind_sub?mode=bfind&max_hit=10000&dbkey=hsa&keywords=DDIT3&mode=bfind)//[DERL2](http://www.genome.jp/dbget-bin/www_bfind_sub?mode=bfind&max_hit=10000&dbkey=hsa&keywords=DERL2&mode=bfind)//[DNAJC1](http://www.genome.jp/dbget-bin/www_bfind_sub?mode=bfind&max_hit=10000&dbkey=hsa&keywords=DNAJC1&mode=bfind)//[ERO1L](http://www.genome.jp/dbget-bin/www_bfind_sub?mode=bfind&max_hit=10000&dbkey=hsa&keywords=ERO1L&mode=bfind)//[HSP90AA1](http://www.genome.jp/dbget-bin/www_bfind_sub?mode=bfind&max_hit=10000&dbkey=hsa&keywords=HSP90AA1&mode=bfind)//[HSPA4L](http://www.genome.jp/dbget-bin/www_bfind_sub?mode=bfind&max_hit=10000&dbkey=hsa&keywords=HSPA4L&mode=bfind)//[HSPA8](http://www.genome.jp/dbget-bin/www_bfind_sub?mode=bfind&max_hit=10000&dbkey=hsa&keywords=HSPA8&mode=bfind)//[LMAN2](http://www.genome.jp/dbget-bin/www_bfind_sub?mode=bfind&max_hit=10000&dbkey=hsa&keywords=LMAN2&mode=bfind)//[SIL1](http://www.genome.jp/dbget-bin/www_bfind_sub?mode=bfind&max_hit=10000&dbkey=hsa&keywords=SIL1&mode=bfind)//[SSR1](http://www.genome.jp/dbget-bin/www_bfind_sub?mode=bfind&max_hit=10000&dbkey=hsa&keywords=SSR1&mode=bfind)//[SSR3](http://www.genome.jp/dbget-bin/www_bfind_sub?mode=bfind&max_hit=10000&dbkey=hsa&keywords=SSR3&mode=bfind)//[STT3B](http://www.genome.jp/dbget-bin/www_bfind_sub?mode=bfind&max_hit=10000&dbkey=hsa&keywords=STT3B&mode=bfind)//[UBE2D2](http://www.genome.jp/dbget-bin/www_bfind_sub?mode=bfind&max_hit=10000&dbkey=hsa&keywords=UBE2D2&mode=bfind)//[UBE2E2](http://www.genome.jp/dbget-bin/www_bfind_sub?mode=bfind&max_hit=10000&dbkey=hsa&keywords=UBE2E2&mode=bfind)//[UBE4B](http://www.genome.jp/dbget-bin/www_bfind_sub?mode=bfind&max_hit=10000&dbkey=hsa&keywords=UBE4B&mode=bfind) |
| [hsa00480](http://www.genome.jp/dbget-bin/show_pathway?hsa00480+2729+79017+2686+2876+221357+2944+6241) | Glutathione metabolism - Homo sapiens (human) | 3.647543e-003 | 7 | 256 | 49 | 6205 | 4.577667e-001 | 2.438000e+000 | [GCLC](http://www.genome.jp/dbget-bin/www_bfind_sub?mode=bfind&max_hit=10000&dbkey=hsa&keywords=GCLC&mode=bfind)//[GGCT](http://www.genome.jp/dbget-bin/www_bfind_sub?mode=bfind&max_hit=10000&dbkey=hsa&keywords=GGCT&mode=bfind)//[GGT7](http://www.genome.jp/dbget-bin/www_bfind_sub?mode=bfind&max_hit=10000&dbkey=hsa&keywords=GGT7&mode=bfind)//[GPX1](http://www.genome.jp/dbget-bin/www_bfind_sub?mode=bfind&max_hit=10000&dbkey=hsa&keywords=GPX1&mode=bfind)//[GSTA5](http://www.genome.jp/dbget-bin/www_bfind_sub?mode=bfind&max_hit=10000&dbkey=hsa&keywords=GSTA5&mode=bfind)//[GSTM1](http://www.genome.jp/dbget-bin/www_bfind_sub?mode=bfind&max_hit=10000&dbkey=hsa&keywords=GSTM1&mode=bfind)//[RRM2](http://www.genome.jp/dbget-bin/www_bfind_sub?mode=bfind&max_hit=10000&dbkey=hsa&keywords=RRM2&mode=bfind) |
| [hsa04722](http://www.genome.jp/dbget-bin/show_pathway?hsa04722+25+397+581+627+2549+2932+3265+11213+3845+4215+4916+7534) | Neurotrophin signaling pathway - Homo sapiens (human) | 5.929535e-003 | 12 | 256 | 127 | 6205 | 4.961044e-001 | 2.226979e+000 | [ABL1](http://www.genome.jp/dbget-bin/www_bfind_sub?mode=bfind&max_hit=10000&dbkey=hsa&keywords=ABL1&mode=bfind)//[ARHGDIB](http://www.genome.jp/dbget-bin/www_bfind_sub?mode=bfind&max_hit=10000&dbkey=hsa&keywords=ARHGDIB&mode=bfind)//[BAX](http://www.genome.jp/dbget-bin/www_bfind_sub?mode=bfind&max_hit=10000&dbkey=hsa&keywords=BAX&mode=bfind)//[BDNF](http://www.genome.jp/dbget-bin/www_bfind_sub?mode=bfind&max_hit=10000&dbkey=hsa&keywords=BDNF&mode=bfind)//[GAB1](http://www.genome.jp/dbget-bin/www_bfind_sub?mode=bfind&max_hit=10000&dbkey=hsa&keywords=GAB1&mode=bfind)//[GSK3B](http://www.genome.jp/dbget-bin/www_bfind_sub?mode=bfind&max_hit=10000&dbkey=hsa&keywords=GSK3B&mode=bfind)//[HRAS](http://www.genome.jp/dbget-bin/www_bfind_sub?mode=bfind&max_hit=10000&dbkey=hsa&keywords=HRAS&mode=bfind)//[IRAK3](http://www.genome.jp/dbget-bin/www_bfind_sub?mode=bfind&max_hit=10000&dbkey=hsa&keywords=IRAK3&mode=bfind)//[KRAS](http://www.genome.jp/dbget-bin/www_bfind_sub?mode=bfind&max_hit=10000&dbkey=hsa&keywords=KRAS&mode=bfind)//[MAP3K3](http://www.genome.jp/dbget-bin/www_bfind_sub?mode=bfind&max_hit=10000&dbkey=hsa&keywords=MAP3K3&mode=bfind)//[NTRK3](http://www.genome.jp/dbget-bin/www_bfind_sub?mode=bfind&max_hit=10000&dbkey=hsa&keywords=NTRK3&mode=bfind)//[YWHAZ](http://www.genome.jp/dbget-bin/www_bfind_sub?mode=bfind&max_hit=10000&dbkey=hsa&keywords=YWHAZ&mode=bfind) |
| [hsa04130](http://www.genome.jp/dbget-bin/show_pathway?hsa04130+9554+8675+6809+6810+8673) | SNARE interactions in vesicular transport - Homo sapiens (human) | 1.520632e-002 | 5 | 256 | 36 | 6205 | 7.716110e-001 | 1.817976e+000 | [SEC22B](http://www.genome.jp/dbget-bin/www_bfind_sub?mode=bfind&max_hit=10000&dbkey=hsa&keywords=SEC22B&mode=bfind)//[STX16](http://www.genome.jp/dbget-bin/www_bfind_sub?mode=bfind&max_hit=10000&dbkey=hsa&keywords=STX16&mode=bfind)//[STX3](http://www.genome.jp/dbget-bin/www_bfind_sub?mode=bfind&max_hit=10000&dbkey=hsa&keywords=STX3&mode=bfind)//[STX4](http://www.genome.jp/dbget-bin/www_bfind_sub?mode=bfind&max_hit=10000&dbkey=hsa&keywords=STX4&mode=bfind)//[VAMP8](http://www.genome.jp/dbget-bin/www_bfind_sub?mode=bfind&max_hit=10000&dbkey=hsa&keywords=VAMP8&mode=bfind) |
| [hsa00983](http://www.genome.jp/dbget-bin/show_pathway?hsa00983+1576+64816+8833+3704+83549+54963) | Drug metabolism - other enzymes - Homo sapiens (human) | 1.766031e-002 | 6 | 256 | 51 | 6205 | 7.716110e-001 | 1.753002e+000 | [CYP3A4](http://www.genome.jp/dbget-bin/www_bfind_sub?mode=bfind&max_hit=10000&dbkey=hsa&keywords=CYP3A4&mode=bfind)//[CYP3A43](http://www.genome.jp/dbget-bin/www_bfind_sub?mode=bfind&max_hit=10000&dbkey=hsa&keywords=CYP3A43&mode=bfind)//[GMPS](http://www.genome.jp/dbget-bin/www_bfind_sub?mode=bfind&max_hit=10000&dbkey=hsa&keywords=GMPS&mode=bfind)//[ITPA](http://www.genome.jp/dbget-bin/www_bfind_sub?mode=bfind&max_hit=10000&dbkey=hsa&keywords=ITPA&mode=bfind)//[UCK1](http://www.genome.jp/dbget-bin/www_bfind_sub?mode=bfind&max_hit=10000&dbkey=hsa&keywords=UCK1&mode=bfind)//[UCKL1](http://www.genome.jp/dbget-bin/www_bfind_sub?mode=bfind&max_hit=10000&dbkey=hsa&keywords=UCKL1&mode=bfind) |
| [hsa05130](http://www.genome.jp/dbget-bin/show_pathway?hsa05130+25+10109+1499+3688+4691+7534) | Pathogenic Escherichia coli infection - Homo sapiens (human) | 2.481843e-002 | 6 | 256 | 55 | 6205 | 7.716110e-001 | 1.605226e+000 | [ABL1](http://www.genome.jp/dbget-bin/www_bfind_sub?mode=bfind&max_hit=10000&dbkey=hsa&keywords=ABL1&mode=bfind)//[ARPC2](http://www.genome.jp/dbget-bin/www_bfind_sub?mode=bfind&max_hit=10000&dbkey=hsa&keywords=ARPC2&mode=bfind)//[CTNNB1](http://www.genome.jp/dbget-bin/www_bfind_sub?mode=bfind&max_hit=10000&dbkey=hsa&keywords=CTNNB1&mode=bfind)//[ITGB1](http://www.genome.jp/dbget-bin/www_bfind_sub?mode=bfind&max_hit=10000&dbkey=hsa&keywords=ITGB1&mode=bfind)//[NCL](http://www.genome.jp/dbget-bin/www_bfind_sub?mode=bfind&max_hit=10000&dbkey=hsa&keywords=NCL&mode=bfind)//[YWHAZ](http://www.genome.jp/dbget-bin/www_bfind_sub?mode=bfind&max_hit=10000&dbkey=hsa&keywords=YWHAZ&mode=bfind) |
| [hsa04012](http://www.genome.jp/dbget-bin/show_pathway?hsa04012+25+2549+2932+3265+3845+3084+10298+6198) | ErbB signaling pathway - Homo sapiens (human) | 2.641968e-002 | 8 | 256 | 87 | 6205 | 7.716110e-001 | 1.578072e+000 | [ABL1](http://www.genome.jp/dbget-bin/www_bfind_sub?mode=bfind&max_hit=10000&dbkey=hsa&keywords=ABL1&mode=bfind)//[GAB1](http://www.genome.jp/dbget-bin/www_bfind_sub?mode=bfind&max_hit=10000&dbkey=hsa&keywords=GAB1&mode=bfind)//[GSK3B](http://www.genome.jp/dbget-bin/www_bfind_sub?mode=bfind&max_hit=10000&dbkey=hsa&keywords=GSK3B&mode=bfind)//[HRAS](http://www.genome.jp/dbget-bin/www_bfind_sub?mode=bfind&max_hit=10000&dbkey=hsa&keywords=HRAS&mode=bfind)//[KRAS](http://www.genome.jp/dbget-bin/www_bfind_sub?mode=bfind&max_hit=10000&dbkey=hsa&keywords=KRAS&mode=bfind)//[NRG1](http://www.genome.jp/dbget-bin/www_bfind_sub?mode=bfind&max_hit=10000&dbkey=hsa&keywords=NRG1&mode=bfind)//[PAK4](http://www.genome.jp/dbget-bin/www_bfind_sub?mode=bfind&max_hit=10000&dbkey=hsa&keywords=PAK4&mode=bfind)//[RPS6KB1](http://www.genome.jp/dbget-bin/www_bfind_sub?mode=bfind&max_hit=10000&dbkey=hsa&keywords=RPS6KB1&mode=bfind) |
| [hsa04340](http://www.genome.jp/dbget-bin/show_pathway?hsa04340+650+654+1452+1456+2932+5727) | Hedgehog signaling pathway - Homo sapiens (human) | 2.687112e-002 | 6 | 256 | 56 | 6205 | 7.716110e-001 | 1.570714e+000 | [BMP2](http://www.genome.jp/dbget-bin/www_bfind_sub?mode=bfind&max_hit=10000&dbkey=hsa&keywords=BMP2&mode=bfind)//[BMP6](http://www.genome.jp/dbget-bin/www_bfind_sub?mode=bfind&max_hit=10000&dbkey=hsa&keywords=BMP6&mode=bfind)//[CSNK1A1](http://www.genome.jp/dbget-bin/www_bfind_sub?mode=bfind&max_hit=10000&dbkey=hsa&keywords=CSNK1A1&mode=bfind)//[CSNK1G3](http://www.genome.jp/dbget-bin/www_bfind_sub?mode=bfind&max_hit=10000&dbkey=hsa&keywords=CSNK1G3&mode=bfind)//[GSK3B](http://www.genome.jp/dbget-bin/www_bfind_sub?mode=bfind&max_hit=10000&dbkey=hsa&keywords=GSK3B&mode=bfind)//[PTCH1](http://www.genome.jp/dbget-bin/www_bfind_sub?mode=bfind&max_hit=10000&dbkey=hsa&keywords=PTCH1&mode=bfind) |
| [hsa00100](http://www.genome.jp/dbget-bin/show_pathway?hsa00100+120227+1718+7108) | Steroid biosynthesis - Homo sapiens (human) | 3.074147e-002 | 3 | 256 | 17 | 6205 | 7.716110e-001 | 1.512275e+000 | [CYP2R1](http://www.genome.jp/dbget-bin/www_bfind_sub?mode=bfind&max_hit=10000&dbkey=hsa&keywords=CYP2R1&mode=bfind)//[DHCR24](http://www.genome.jp/dbget-bin/www_bfind_sub?mode=bfind&max_hit=10000&dbkey=hsa&keywords=DHCR24&mode=bfind)//[TM7SF2](http://www.genome.jp/dbget-bin/www_bfind_sub?mode=bfind&max_hit=10000&dbkey=hsa&keywords=TM7SF2&mode=bfind) |
| [hsa00770](http://www.genome.jp/dbget-bin/show_pathway?hsa00770+60496+53354+79646) | Pantothenate and CoA biosynthesis - Homo sapiens (human) | 3.074147e-002 | 3 | 256 | 17 | 6205 | 7.716110e-001 | 1.512275e+000 | [AASDHPPT](http://www.genome.jp/dbget-bin/www_bfind_sub?mode=bfind&max_hit=10000&dbkey=hsa&keywords=AASDHPPT&mode=bfind)//[PANK1](http://www.genome.jp/dbget-bin/www_bfind_sub?mode=bfind&max_hit=10000&dbkey=hsa&keywords=PANK1&mode=bfind)//[PANK3](http://www.genome.jp/dbget-bin/www_bfind_sub?mode=bfind&max_hit=10000&dbkey=hsa&keywords=PANK3&mode=bfind) |
| [hsa00512](http://www.genome.jp/dbget-bin/show_pathway?hsa00512+51809+26290+57452+2650) | Mucin type O-Glycan biosynthesis - Homo sapiens (human) | 3.710426e-002 | 4 | 256 | 31 | 6205 | 8.466518e-001 | 1.430576e+000 | [GALNT7](http://www.genome.jp/dbget-bin/www_bfind_sub?mode=bfind&max_hit=10000&dbkey=hsa&keywords=GALNT7&mode=bfind)//[GALNT8](http://www.genome.jp/dbget-bin/www_bfind_sub?mode=bfind&max_hit=10000&dbkey=hsa&keywords=GALNT8&mode=bfind)//[GALNTL1](http://www.genome.jp/dbget-bin/www_bfind_sub?mode=bfind&max_hit=10000&dbkey=hsa&keywords=GALNTL1&mode=bfind)//[GCNT1](http://www.genome.jp/dbget-bin/www_bfind_sub?mode=bfind&max_hit=10000&dbkey=hsa&keywords=GCNT1&mode=bfind) |

#### Mimic-vs-Control (down)

| **PathwayID** | **Definition** | **Fisher-Pvalue** | **SelectionCounts** | **SelectionSize** | **Count** | **Size** | **FDR** | **Enrichment_Score** | **Genes** |
| --- | --- | --- | --- | --- | --- | --- | --- | --- | --- |
| [hsa00983](http://www.genome.jp/dbget-bin/show_pathway?hsa00983+1576+64816+1890+54963+54578) | Drug metabolism - other enzymes - Homo sapiens (human) | 2.572807e-003 | 5 | 117 | 51 | 6205 | 4.873054e-001 | 2.589593e+000 | [CYP3A4](http://www.genome.jp/dbget-bin/www_bfind_sub?mode=bfind&max_hit=10000&dbkey=hsa&keywords=CYP3A4&mode=bfind)//[CYP3A43](http://www.genome.jp/dbget-bin/www_bfind_sub?mode=bfind&max_hit=10000&dbkey=hsa&keywords=CYP3A43&mode=bfind)//[TYMP](http://www.genome.jp/dbget-bin/www_bfind_sub?mode=bfind&max_hit=10000&dbkey=hsa&keywords=TYMP&mode=bfind)//[UCKL1](http://www.genome.jp/dbget-bin/www_bfind_sub?mode=bfind&max_hit=10000&dbkey=hsa&keywords=UCKL1&mode=bfind)//[UGT1A6](http://www.genome.jp/dbget-bin/www_bfind_sub?mode=bfind&max_hit=10000&dbkey=hsa&keywords=UGT1A6&mode=bfind) |
| [hsa04340](http://www.genome.jp/dbget-bin/show_pathway?hsa04340+650+1452+1456+2932+5568) | Hedgehog signaling pathway - Homo sapiens (human) | 3.882912e-003 | 5 | 117 | 56 | 6205 | 4.873054e-001 | 2.410842e+000 | [BMP2](http://www.genome.jp/dbget-bin/www_bfind_sub?mode=bfind&max_hit=10000&dbkey=hsa&keywords=BMP2&mode=bfind)//[CSNK1A1](http://www.genome.jp/dbget-bin/www_bfind_sub?mode=bfind&max_hit=10000&dbkey=hsa&keywords=CSNK1A1&mode=bfind)//[CSNK1G3](http://www.genome.jp/dbget-bin/www_bfind_sub?mode=bfind&max_hit=10000&dbkey=hsa&keywords=CSNK1G3&mode=bfind)//[GSK3B](http://www.genome.jp/dbget-bin/www_bfind_sub?mode=bfind&max_hit=10000&dbkey=hsa&keywords=GSK3B&mode=bfind)//[PRKACG](http://www.genome.jp/dbget-bin/www_bfind_sub?mode=bfind&max_hit=10000&dbkey=hsa&keywords=PRKACG&mode=bfind) |
| [hsa05414](http://www.genome.jp/dbget-bin/show_pathway?hsa05414+59285+3655+3688+5568+6546+7168) | Dilated cardiomyopathy - Homo sapiens (human) | 7.550716e-003 | 6 | 117 | 92 | 6205 | 5.919524e-001 | 2.122012e+000 | [CACNG6](http://www.genome.jp/dbget-bin/www_bfind_sub?mode=bfind&max_hit=10000&dbkey=hsa&keywords=CACNG6&mode=bfind)//[ITGA6](http://www.genome.jp/dbget-bin/www_bfind_sub?mode=bfind&max_hit=10000&dbkey=hsa&keywords=ITGA6&mode=bfind)//[ITGB1](http://www.genome.jp/dbget-bin/www_bfind_sub?mode=bfind&max_hit=10000&dbkey=hsa&keywords=ITGB1&mode=bfind)//[PRKACG](http://www.genome.jp/dbget-bin/www_bfind_sub?mode=bfind&max_hit=10000&dbkey=hsa&keywords=PRKACG&mode=bfind)//[SLC8A1](http://www.genome.jp/dbget-bin/www_bfind_sub?mode=bfind&max_hit=10000&dbkey=hsa&keywords=SLC8A1&mode=bfind)//[TPM1](http://www.genome.jp/dbget-bin/www_bfind_sub?mode=bfind&max_hit=10000&dbkey=hsa&keywords=TPM1&mode=bfind) |
| [hsa04724](http://www.genome.jp/dbget-bin/show_pathway?hsa04724+54331+2905+5321+5530+5568+246213+6507) | Glutamatergic synapse - Homo sapiens (human) | 9.433504e-003 | 7 | 117 | 126 | 6205 | 5.919524e-001 | 2.025327e+000 | [GNG2](http://www.genome.jp/dbget-bin/www_bfind_sub?mode=bfind&max_hit=10000&dbkey=hsa&keywords=GNG2&mode=bfind)//[GRIN2C](http://www.genome.jp/dbget-bin/www_bfind_sub?mode=bfind&max_hit=10000&dbkey=hsa&keywords=GRIN2C&mode=bfind)//[PLA2G4A](http://www.genome.jp/dbget-bin/www_bfind_sub?mode=bfind&max_hit=10000&dbkey=hsa&keywords=PLA2G4A&mode=bfind)//[PPP3CA](http://www.genome.jp/dbget-bin/www_bfind_sub?mode=bfind&max_hit=10000&dbkey=hsa&keywords=PPP3CA&mode=bfind)//[PRKACG](http://www.genome.jp/dbget-bin/www_bfind_sub?mode=bfind&max_hit=10000&dbkey=hsa&keywords=PRKACG&mode=bfind)//[SLC17A8](http://www.genome.jp/dbget-bin/www_bfind_sub?mode=bfind&max_hit=10000&dbkey=hsa&keywords=SLC17A8&mode=bfind)//[SLC1A3](http://www.genome.jp/dbget-bin/www_bfind_sub?mode=bfind&max_hit=10000&dbkey=hsa&keywords=SLC1A3&mode=bfind) |
| [hsa04961](http://www.genome.jp/dbget-bin/show_pathway?hsa04961+483+3817+5568+6546) | Endocrine and other factor-regulated calcium reabsorption - Homo sapiens (human) | 1.324537e-002 | 4 | 117 | 49 | 6205 | 6.649177e-001 | 1.877936e+000 | [ATP1B3](http://www.genome.jp/dbget-bin/www_bfind_sub?mode=bfind&max_hit=10000&dbkey=hsa&keywords=ATP1B3&mode=bfind)//[KLK2](http://www.genome.jp/dbget-bin/www_bfind_sub?mode=bfind&max_hit=10000&dbkey=hsa&keywords=KLK2&mode=bfind)//[PRKACG](http://www.genome.jp/dbget-bin/www_bfind_sub?mode=bfind&max_hit=10000&dbkey=hsa&keywords=PRKACG&mode=bfind)//[SLC8A1](http://www.genome.jp/dbget-bin/www_bfind_sub?mode=bfind&max_hit=10000&dbkey=hsa&keywords=SLC8A1&mode=bfind) |
| [hsa00591](http://www.genome.jp/dbget-bin/show_pathway?hsa00591+1576+64816+5321) | Linoleic acid metabolism - Homo sapiens (human) | 1.671038e-002 | 3 | 117 | 29 | 6205 | 6.990509e-001 | 1.777014e+000 | [CYP3A4](http://www.genome.jp/dbget-bin/www_bfind_sub?mode=bfind&max_hit=10000&dbkey=hsa&keywords=CYP3A4&mode=bfind)//[CYP3A43](http://www.genome.jp/dbget-bin/www_bfind_sub?mode=bfind&max_hit=10000&dbkey=hsa&keywords=CYP3A43&mode=bfind)//[PLA2G4A](http://www.genome.jp/dbget-bin/www_bfind_sub?mode=bfind&max_hit=10000&dbkey=hsa&keywords=PLA2G4A&mode=bfind) |
| [hsa05410](http://www.genome.jp/dbget-bin/show_pathway?hsa05410+59285+3655+3688+6546+7168) | Hypertrophic cardiomyopathy (HCM) - Homo sapiens (human) | 1.977992e-002 | 5 | 117 | 83 | 6205 | 7.092516e-001 | 1.703775e+000 | [CACNG6](http://www.genome.jp/dbget-bin/www_bfind_sub?mode=bfind&max_hit=10000&dbkey=hsa&keywords=CACNG6&mode=bfind)//[ITGA6](http://www.genome.jp/dbget-bin/www_bfind_sub?mode=bfind&max_hit=10000&dbkey=hsa&keywords=ITGA6&mode=bfind)//[ITGB1](http://www.genome.jp/dbget-bin/www_bfind_sub?mode=bfind&max_hit=10000&dbkey=hsa&keywords=ITGB1&mode=bfind)//[SLC8A1](http://www.genome.jp/dbget-bin/www_bfind_sub?mode=bfind&max_hit=10000&dbkey=hsa&keywords=SLC8A1&mode=bfind)//[TPM1](http://www.genome.jp/dbget-bin/www_bfind_sub?mode=bfind&max_hit=10000&dbkey=hsa&keywords=TPM1&mode=bfind) |
| [hsa04360](http://www.genome.jp/dbget-bin/show_pathway?hsa04360+2932+3688+4772+5530+5921+10371) | Axon guidance - Homo sapiens (human) | 3.450045e-002 | 6 | 117 | 129 | 6205 | 7.908189e-001 | 1.462175e+000 | [GSK3B](http://www.genome.jp/dbget-bin/www_bfind_sub?mode=bfind&max_hit=10000&dbkey=hsa&keywords=GSK3B&mode=bfind)//[ITGB1](http://www.genome.jp/dbget-bin/www_bfind_sub?mode=bfind&max_hit=10000&dbkey=hsa&keywords=ITGB1&mode=bfind)//[NFATC1](http://www.genome.jp/dbget-bin/www_bfind_sub?mode=bfind&max_hit=10000&dbkey=hsa&keywords=NFATC1&mode=bfind)//[PPP3CA](http://www.genome.jp/dbget-bin/www_bfind_sub?mode=bfind&max_hit=10000&dbkey=hsa&keywords=PPP3CA&mode=bfind)//[RASA1](http://www.genome.jp/dbget-bin/www_bfind_sub?mode=bfind&max_hit=10000&dbkey=hsa&keywords=RASA1&mode=bfind)//[SEMA3A](http://www.genome.jp/dbget-bin/www_bfind_sub?mode=bfind&max_hit=10000&dbkey=hsa&keywords=SEMA3A&mode=bfind) |
| [hsa00830](http://www.genome.jp/dbget-bin/show_pathway?hsa00830+1576+64816+10901+54578) | Retinol metabolism - Homo sapiens (human) | 3.535799e-002 | 4 | 117 | 66 | 6205 | 7.908189e-001 | 1.451512e+000 | [CYP3A4](http://www.genome.jp/dbget-bin/www_bfind_sub?mode=bfind&max_hit=10000&dbkey=hsa&keywords=CYP3A4&mode=bfind)//[CYP3A43](http://www.genome.jp/dbget-bin/www_bfind_sub?mode=bfind&max_hit=10000&dbkey=hsa&keywords=CYP3A43&mode=bfind)//[DHRS4](http://www.genome.jp/dbget-bin/www_bfind_sub?mode=bfind&max_hit=10000&dbkey=hsa&keywords=DHRS4&mode=bfind)//[UGT1A6](http://www.genome.jp/dbget-bin/www_bfind_sub?mode=bfind&max_hit=10000&dbkey=hsa&keywords=UGT1A6&mode=bfind) |
| [hsa04728](http://www.genome.jp/dbget-bin/show_pathway?hsa04728+409+1386+54331+2932+5530+5568) | Dopaminergic synapse - Homo sapiens (human) | 3.563270e-002 | 6 | 117 | 130 | 6205 | 7.908189e-001 | 1.448151e+000 | [ARRB2](http://www.genome.jp/dbget-bin/www_bfind_sub?mode=bfind&max_hit=10000&dbkey=hsa&keywords=ARRB2&mode=bfind)//[ATF2](http://www.genome.jp/dbget-bin/www_bfind_sub?mode=bfind&max_hit=10000&dbkey=hsa&keywords=ATF2&mode=bfind)//[GNG2](http://www.genome.jp/dbget-bin/www_bfind_sub?mode=bfind&max_hit=10000&dbkey=hsa&keywords=GNG2&mode=bfind)//[GSK3B](http://www.genome.jp/dbget-bin/www_bfind_sub?mode=bfind&max_hit=10000&dbkey=hsa&keywords=GSK3B&mode=bfind)//[PPP3CA](http://www.genome.jp/dbget-bin/www_bfind_sub?mode=bfind&max_hit=10000&dbkey=hsa&keywords=PPP3CA&mode=bfind)//[PRKACG](http://www.genome.jp/dbget-bin/www_bfind_sub?mode=bfind&max_hit=10000&dbkey=hsa&keywords=PRKACG&mode=bfind) |
| [hsa04614](http://www.genome.jp/dbget-bin/show_pathway?hsa04614+5476+4311) | Renin-angiotensin system - Homo sapiens (human) | 3.986460e-002 | 2 | 117 | 17 | 6205 | 7.908189e-001 | 1.399413e+000 | [CTSA](http://www.genome.jp/dbget-bin/www_bfind_sub?mode=bfind&max_hit=10000&dbkey=hsa&keywords=CTSA&mode=bfind)//[MME](http://www.genome.jp/dbget-bin/www_bfind_sub?mode=bfind&max_hit=10000&dbkey=hsa&keywords=MME&mode=bfind) |
| [hsa05031](http://www.genome.jp/dbget-bin/show_pathway?hsa05031+1386+2905+5530+5568) | Amphetamine addiction - Homo sapiens (human) | 4.250514e-002 | 4 | 117 | 70 | 6205 | 7.908189e-001 | 1.371559e+000 | [ATF2](http://www.genome.jp/dbget-bin/www_bfind_sub?mode=bfind&max_hit=10000&dbkey=hsa&keywords=ATF2&mode=bfind)//[GRIN2C](http://www.genome.jp/dbget-bin/www_bfind_sub?mode=bfind&max_hit=10000&dbkey=hsa&keywords=GRIN2C&mode=bfind)//[PPP3CA](http://www.genome.jp/dbget-bin/www_bfind_sub?mode=bfind&max_hit=10000&dbkey=hsa&keywords=PPP3CA&mode=bfind)//[PRKACG](http://www.genome.jp/dbget-bin/www_bfind_sub?mode=bfind&max_hit=10000&dbkey=hsa&keywords=PRKACG&mode=bfind) |
| [hsa04910](http://www.genome.jp/dbget-bin/show_pathway?hsa04910+23265+2932+2997+8569+5568+8651) | Insulin signaling pathway - Homo sapiens (human) | 4.555718e-002 | 6 | 117 | 138 | 6205 | 7.908189e-001 | 1.341443e+000 | [EXOC7](http://www.genome.jp/dbget-bin/www_bfind_sub?mode=bfind&max_hit=10000&dbkey=hsa&keywords=EXOC7&mode=bfind)//[GSK3B](http://www.genome.jp/dbget-bin/www_bfind_sub?mode=bfind&max_hit=10000&dbkey=hsa&keywords=GSK3B&mode=bfind)//[GYS1](http://www.genome.jp/dbget-bin/www_bfind_sub?mode=bfind&max_hit=10000&dbkey=hsa&keywords=GYS1&mode=bfind)//[MKNK1](http://www.genome.jp/dbget-bin/www_bfind_sub?mode=bfind&max_hit=10000&dbkey=hsa&keywords=MKNK1&mode=bfind)//[PRKACG](http://www.genome.jp/dbget-bin/www_bfind_sub?mode=bfind&max_hit=10000&dbkey=hsa&keywords=PRKACG&mode=bfind)//[SOCS1](http://www.genome.jp/dbget-bin/www_bfind_sub?mode=bfind&max_hit=10000&dbkey=hsa&keywords=SOCS1&mode=bfind) |
| [hsa00982](http://www.genome.jp/dbget-bin/show_pathway?hsa00982+1576+64816+2944+54578) | Drug metabolism - cytochrome P450 - Homo sapiens (human) | 4.636325e-002 | 4 | 117 | 72 | 6205 | 7.908189e-001 | 1.333826e+000 | [CYP3A4](http://www.genome.jp/dbget-bin/www_bfind_sub?mode=bfind&max_hit=10000&dbkey=hsa&keywords=CYP3A4&mode=bfind)//[CYP3A43](http://www.genome.jp/dbget-bin/www_bfind_sub?mode=bfind&max_hit=10000&dbkey=hsa&keywords=CYP3A43&mode=bfind)//[GSTM1](http://www.genome.jp/dbget-bin/www_bfind_sub?mode=bfind&max_hit=10000&dbkey=hsa&keywords=GSTM1&mode=bfind)//[UGT1A6](http://www.genome.jp/dbget-bin/www_bfind_sub?mode=bfind&max_hit=10000&dbkey=hsa&keywords=UGT1A6&mode=bfind) |
| [hsa04962](http://www.genome.jp/dbget-bin/show_pathway?hsa04962+397+4905+5568) | Vasopressin-regulated water reabsorption - Homo sapiens (human) | 4.947539e-002 | 3 | 117 | 44 | 6205 | 7.908189e-001 | 1.305611e+000 | [ARHGDIB](http://www.genome.jp/dbget-bin/www_bfind_sub?mode=bfind&max_hit=10000&dbkey=hsa&keywords=ARHGDIB&mode=bfind)//[NSF](http://www.genome.jp/dbget-bin/www_bfind_sub?mode=bfind&max_hit=10000&dbkey=hsa&keywords=NSF&mode=bfind)//[PRKACG](http://www.genome.jp/dbget-bin/www_bfind_sub?mode=bfind&max_hit=10000&dbkey=hsa&keywords=PRKACG&mode=bfind) |

#### H77-vs-Control (up)

| **PathwayID** | **Definition** | **Fisher-Pvalue** | **SelectionCounts** | **SelectionSize** | **Count** | **Size** | **FDR** | **Enrichment_Score** | **Genes** |
| --- | --- | --- | --- | --- | --- | --- | --- | --- | --- |
| [hsa04141](http://www.genome.jp/dbget-bin/show_pathway?hsa04141+573+581+821+1649+51009+64215+30001+3320+22824+3312+10960+64374+6745+6747+201595+7322+7325+10277) | Protein processing in endoplasmic reticulum - Homo sapiens (human) | 1.493082e-004 | 18 | 256 | 166 | 6205 | 3.747637e-002 | 3.825916e+000 | [BAG1](http://www.genome.jp/dbget-bin/www_bfind_sub?mode=bfind&max_hit=10000&dbkey=hsa&keywords=BAG1&mode=bfind)//[BAX](http://www.genome.jp/dbget-bin/www_bfind_sub?mode=bfind&max_hit=10000&dbkey=hsa&keywords=BAX&mode=bfind)//[CANX](http://www.genome.jp/dbget-bin/www_bfind_sub?mode=bfind&max_hit=10000&dbkey=hsa&keywords=CANX&mode=bfind)//[DDIT3](http://www.genome.jp/dbget-bin/www_bfind_sub?mode=bfind&max_hit=10000&dbkey=hsa&keywords=DDIT3&mode=bfind)//[DERL2](http://www.genome.jp/dbget-bin/www_bfind_sub?mode=bfind&max_hit=10000&dbkey=hsa&keywords=DERL2&mode=bfind)//[DNAJC1](http://www.genome.jp/dbget-bin/www_bfind_sub?mode=bfind&max_hit=10000&dbkey=hsa&keywords=DNAJC1&mode=bfind)//[ERO1L](http://www.genome.jp/dbget-bin/www_bfind_sub?mode=bfind&max_hit=10000&dbkey=hsa&keywords=ERO1L&mode=bfind)//[HSP90AA1](http://www.genome.jp/dbget-bin/www_bfind_sub?mode=bfind&max_hit=10000&dbkey=hsa&keywords=HSP90AA1&mode=bfind)//[HSPA4L](http://www.genome.jp/dbget-bin/www_bfind_sub?mode=bfind&max_hit=10000&dbkey=hsa&keywords=HSPA4L&mode=bfind)//[HSPA8](http://www.genome.jp/dbget-bin/www_bfind_sub?mode=bfind&max_hit=10000&dbkey=hsa&keywords=HSPA8&mode=bfind)//[LMAN2](http://www.genome.jp/dbget-bin/www_bfind_sub?mode=bfind&max_hit=10000&dbkey=hsa&keywords=LMAN2&mode=bfind)//[SIL1](http://www.genome.jp/dbget-bin/www_bfind_sub?mode=bfind&max_hit=10000&dbkey=hsa&keywords=SIL1&mode=bfind)//[SSR1](http://www.genome.jp/dbget-bin/www_bfind_sub?mode=bfind&max_hit=10000&dbkey=hsa&keywords=SSR1&mode=bfind)//[SSR3](http://www.genome.jp/dbget-bin/www_bfind_sub?mode=bfind&max_hit=10000&dbkey=hsa&keywords=SSR3&mode=bfind)//[STT3B](http://www.genome.jp/dbget-bin/www_bfind_sub?mode=bfind&max_hit=10000&dbkey=hsa&keywords=STT3B&mode=bfind)//[UBE2D2](http://www.genome.jp/dbget-bin/www_bfind_sub?mode=bfind&max_hit=10000&dbkey=hsa&keywords=UBE2D2&mode=bfind)//[UBE2E2](http://www.genome.jp/dbget-bin/www_bfind_sub?mode=bfind&max_hit=10000&dbkey=hsa&keywords=UBE2E2&mode=bfind)//[UBE4B](http://www.genome.jp/dbget-bin/www_bfind_sub?mode=bfind&max_hit=10000&dbkey=hsa&keywords=UBE4B&mode=bfind) |
| [hsa00480](http://www.genome.jp/dbget-bin/show_pathway?hsa00480+2729+79017+2686+2876+221357+2944+6241) | Glutathione metabolism - Homo sapiens (human) | 3.647543e-003 | 7 | 256 | 49 | 6205 | 4.577667e-001 | 2.438000e+000 | [GCLC](http://www.genome.jp/dbget-bin/www_bfind_sub?mode=bfind&max_hit=10000&dbkey=hsa&keywords=GCLC&mode=bfind)//[GGCT](http://www.genome.jp/dbget-bin/www_bfind_sub?mode=bfind&max_hit=10000&dbkey=hsa&keywords=GGCT&mode=bfind)//[GGT7](http://www.genome.jp/dbget-bin/www_bfind_sub?mode=bfind&max_hit=10000&dbkey=hsa&keywords=GGT7&mode=bfind)//[GPX1](http://www.genome.jp/dbget-bin/www_bfind_sub?mode=bfind&max_hit=10000&dbkey=hsa&keywords=GPX1&mode=bfind)//[GSTA5](http://www.genome.jp/dbget-bin/www_bfind_sub?mode=bfind&max_hit=10000&dbkey=hsa&keywords=GSTA5&mode=bfind)//[GSTM1](http://www.genome.jp/dbget-bin/www_bfind_sub?mode=bfind&max_hit=10000&dbkey=hsa&keywords=GSTM1&mode=bfind)//[RRM2](http://www.genome.jp/dbget-bin/www_bfind_sub?mode=bfind&max_hit=10000&dbkey=hsa&keywords=RRM2&mode=bfind) |
| [hsa04722](http://www.genome.jp/dbget-bin/show_pathway?hsa04722+25+397+581+627+2549+2932+3265+11213+3845+4215+4916+7534) | Neurotrophin signaling pathway - Homo sapiens (human) | 5.929535e-003 | 12 | 256 | 127 | 6205 | 4.961044e-001 | 2.226979e+000 | [ABL1](http://www.genome.jp/dbget-bin/www_bfind_sub?mode=bfind&max_hit=10000&dbkey=hsa&keywords=ABL1&mode=bfind)//[ARHGDIB](http://www.genome.jp/dbget-bin/www_bfind_sub?mode=bfind&max_hit=10000&dbkey=hsa&keywords=ARHGDIB&mode=bfind)//[BAX](http://www.genome.jp/dbget-bin/www_bfind_sub?mode=bfind&max_hit=10000&dbkey=hsa&keywords=BAX&mode=bfind)//[BDNF](http://www.genome.jp/dbget-bin/www_bfind_sub?mode=bfind&max_hit=10000&dbkey=hsa&keywords=BDNF&mode=bfind)//[GAB1](http://www.genome.jp/dbget-bin/www_bfind_sub?mode=bfind&max_hit=10000&dbkey=hsa&keywords=GAB1&mode=bfind)//[GSK3B](http://www.genome.jp/dbget-bin/www_bfind_sub?mode=bfind&max_hit=10000&dbkey=hsa&keywords=GSK3B&mode=bfind)//[HRAS](http://www.genome.jp/dbget-bin/www_bfind_sub?mode=bfind&max_hit=10000&dbkey=hsa&keywords=HRAS&mode=bfind)//[IRAK3](http://www.genome.jp/dbget-bin/www_bfind_sub?mode=bfind&max_hit=10000&dbkey=hsa&keywords=IRAK3&mode=bfind)//[KRAS](http://www.genome.jp/dbget-bin/www_bfind_sub?mode=bfind&max_hit=10000&dbkey=hsa&keywords=KRAS&mode=bfind)//[MAP3K3](http://www.genome.jp/dbget-bin/www_bfind_sub?mode=bfind&max_hit=10000&dbkey=hsa&keywords=MAP3K3&mode=bfind)//[NTRK3](http://www.genome.jp/dbget-bin/www_bfind_sub?mode=bfind&max_hit=10000&dbkey=hsa&keywords=NTRK3&mode=bfind)//[YWHAZ](http://www.genome.jp/dbget-bin/www_bfind_sub?mode=bfind&max_hit=10000&dbkey=hsa&keywords=YWHAZ&mode=bfind) |
| [hsa04130](http://www.genome.jp/dbget-bin/show_pathway?hsa04130+9554+8675+6809+6810+8673) | SNARE interactions in vesicular transport - Homo sapiens (human) | 1.520632e-002 | 5 | 256 | 36 | 6205 | 7.716110e-001 | 1.817976e+000 | [SEC22B](http://www.genome.jp/dbget-bin/www_bfind_sub?mode=bfind&max_hit=10000&dbkey=hsa&keywords=SEC22B&mode=bfind)//[STX16](http://www.genome.jp/dbget-bin/www_bfind_sub?mode=bfind&max_hit=10000&dbkey=hsa&keywords=STX16&mode=bfind)//[STX3](http://www.genome.jp/dbget-bin/www_bfind_sub?mode=bfind&max_hit=10000&dbkey=hsa&keywords=STX3&mode=bfind)//[STX4](http://www.genome.jp/dbget-bin/www_bfind_sub?mode=bfind&max_hit=10000&dbkey=hsa&keywords=STX4&mode=bfind)//[VAMP8](http://www.genome.jp/dbget-bin/www_bfind_sub?mode=bfind&max_hit=10000&dbkey=hsa&keywords=VAMP8&mode=bfind) |
| [hsa00983](http://www.genome.jp/dbget-bin/show_pathway?hsa00983+1576+64816+8833+3704+83549+54963) | Drug metabolism - other enzymes - Homo sapiens (human) | 1.766031e-002 | 6 | 256 | 51 | 6205 | 7.716110e-001 | 1.753002e+000 | [CYP3A4](http://www.genome.jp/dbget-bin/www_bfind_sub?mode=bfind&max_hit=10000&dbkey=hsa&keywords=CYP3A4&mode=bfind)//[CYP3A43](http://www.genome.jp/dbget-bin/www_bfind_sub?mode=bfind&max_hit=10000&dbkey=hsa&keywords=CYP3A43&mode=bfind)//[GMPS](http://www.genome.jp/dbget-bin/www_bfind_sub?mode=bfind&max_hit=10000&dbkey=hsa&keywords=GMPS&mode=bfind)//[ITPA](http://www.genome.jp/dbget-bin/www_bfind_sub?mode=bfind&max_hit=10000&dbkey=hsa&keywords=ITPA&mode=bfind)//[UCK1](http://www.genome.jp/dbget-bin/www_bfind_sub?mode=bfind&max_hit=10000&dbkey=hsa&keywords=UCK1&mode=bfind)//[UCKL1](http://www.genome.jp/dbget-bin/www_bfind_sub?mode=bfind&max_hit=10000&dbkey=hsa&keywords=UCKL1&mode=bfind) |
| [hsa05130](http://www.genome.jp/dbget-bin/show_pathway?hsa05130+25+10109+1499+3688+4691+7534) | Pathogenic Escherichia coli infection - Homo sapiens (human) | 2.481843e-002 | 6 | 256 | 55 | 6205 | 7.716110e-001 | 1.605226e+000 | [ABL1](http://www.genome.jp/dbget-bin/www_bfind_sub?mode=bfind&max_hit=10000&dbkey=hsa&keywords=ABL1&mode=bfind)//[ARPC2](http://www.genome.jp/dbget-bin/www_bfind_sub?mode=bfind&max_hit=10000&dbkey=hsa&keywords=ARPC2&mode=bfind)//[CTNNB1](http://www.genome.jp/dbget-bin/www_bfind_sub?mode=bfind&max_hit=10000&dbkey=hsa&keywords=CTNNB1&mode=bfind)//[ITGB1](http://www.genome.jp/dbget-bin/www_bfind_sub?mode=bfind&max_hit=10000&dbkey=hsa&keywords=ITGB1&mode=bfind)//[NCL](http://www.genome.jp/dbget-bin/www_bfind_sub?mode=bfind&max_hit=10000&dbkey=hsa&keywords=NCL&mode=bfind)//[YWHAZ](http://www.genome.jp/dbget-bin/www_bfind_sub?mode=bfind&max_hit=10000&dbkey=hsa&keywords=YWHAZ&mode=bfind) |
| [hsa04012](http://www.genome.jp/dbget-bin/show_pathway?hsa04012+25+2549+2932+3265+3845+3084+10298+6198) | ErbB signaling pathway - Homo sapiens (human) | 2.641968e-002 | 8 | 256 | 87 | 6205 | 7.716110e-001 | 1.578072e+000 | [ABL1](http://www.genome.jp/dbget-bin/www_bfind_sub?mode=bfind&max_hit=10000&dbkey=hsa&keywords=ABL1&mode=bfind)//[GAB1](http://www.genome.jp/dbget-bin/www_bfind_sub?mode=bfind&max_hit=10000&dbkey=hsa&keywords=GAB1&mode=bfind)//[GSK3B](http://www.genome.jp/dbget-bin/www_bfind_sub?mode=bfind&max_hit=10000&dbkey=hsa&keywords=GSK3B&mode=bfind)//[HRAS](http://www.genome.jp/dbget-bin/www_bfind_sub?mode=bfind&max_hit=10000&dbkey=hsa&keywords=HRAS&mode=bfind)//[KRAS](http://www.genome.jp/dbget-bin/www_bfind_sub?mode=bfind&max_hit=10000&dbkey=hsa&keywords=KRAS&mode=bfind)//[NRG1](http://www.genome.jp/dbget-bin/www_bfind_sub?mode=bfind&max_hit=10000&dbkey=hsa&keywords=NRG1&mode=bfind)//[PAK4](http://www.genome.jp/dbget-bin/www_bfind_sub?mode=bfind&max_hit=10000&dbkey=hsa&keywords=PAK4&mode=bfind)//[RPS6KB1](http://www.genome.jp/dbget-bin/www_bfind_sub?mode=bfind&max_hit=10000&dbkey=hsa&keywords=RPS6KB1&mode=bfind) |
| [hsa04340](http://www.genome.jp/dbget-bin/show_pathway?hsa04340+650+654+1452+1456+2932+5727) | Hedgehog signaling pathway - Homo sapiens (human) | 2.687112e-002 | 6 | 256 | 56 | 6205 | 7.716110e-001 | 1.570714e+000 | [BMP2](http://www.genome.jp/dbget-bin/www_bfind_sub?mode=bfind&max_hit=10000&dbkey=hsa&keywords=BMP2&mode=bfind)//[BMP6](http://www.genome.jp/dbget-bin/www_bfind_sub?mode=bfind&max_hit=10000&dbkey=hsa&keywords=BMP6&mode=bfind)//[CSNK1A1](http://www.genome.jp/dbget-bin/www_bfind_sub?mode=bfind&max_hit=10000&dbkey=hsa&keywords=CSNK1A1&mode=bfind)//[CSNK1G3](http://www.genome.jp/dbget-bin/www_bfind_sub?mode=bfind&max_hit=10000&dbkey=hsa&keywords=CSNK1G3&mode=bfind)//[GSK3B](http://www.genome.jp/dbget-bin/www_bfind_sub?mode=bfind&max_hit=10000&dbkey=hsa&keywords=GSK3B&mode=bfind)//[PTCH1](http://www.genome.jp/dbget-bin/www_bfind_sub?mode=bfind&max_hit=10000&dbkey=hsa&keywords=PTCH1&mode=bfind) |
| [hsa00100](http://www.genome.jp/dbget-bin/show_pathway?hsa00100+120227+1718+7108) | Steroid biosynthesis - Homo sapiens (human) | 3.074147e-002 | 3 | 256 | 17 | 6205 | 7.716110e-001 | 1.512275e+000 | [CYP2R1](http://www.genome.jp/dbget-bin/www_bfind_sub?mode=bfind&max_hit=10000&dbkey=hsa&keywords=CYP2R1&mode=bfind)//[DHCR24](http://www.genome.jp/dbget-bin/www_bfind_sub?mode=bfind&max_hit=10000&dbkey=hsa&keywords=DHCR24&mode=bfind)//[TM7SF2](http://www.genome.jp/dbget-bin/www_bfind_sub?mode=bfind&max_hit=10000&dbkey=hsa&keywords=TM7SF2&mode=bfind) |
| [hsa00770](http://www.genome.jp/dbget-bin/show_pathway?hsa00770+60496+53354+79646) | Pantothenate and CoA biosynthesis - Homo sapiens (human) | 3.074147e-002 | 3 | 256 | 17 | 6205 | 7.716110e-001 | 1.512275e+000 | [AASDHPPT](http://www.genome.jp/dbget-bin/www_bfind_sub?mode=bfind&max_hit=10000&dbkey=hsa&keywords=AASDHPPT&mode=bfind)//[PANK1](http://www.genome.jp/dbget-bin/www_bfind_sub?mode=bfind&max_hit=10000&dbkey=hsa&keywords=PANK1&mode=bfind)//[PANK3](http://www.genome.jp/dbget-bin/www_bfind_sub?mode=bfind&max_hit=10000&dbkey=hsa&keywords=PANK3&mode=bfind) |
| [hsa00512](http://www.genome.jp/dbget-bin/show_pathway?hsa00512+51809+26290+57452+2650) | Mucin type O-Glycan biosynthesis - Homo sapiens (human) | 3.710426e-002 | 4 | 256 | 31 | 6205 | 8.466518e-001 | 1.430576e+000 | [GALNT7](http://www.genome.jp/dbget-bin/www_bfind_sub?mode=bfind&max_hit=10000&dbkey=hsa&keywords=GALNT7&mode=bfind)//[GALNT8](http://www.genome.jp/dbget-bin/www_bfind_sub?mode=bfind&max_hit=10000&dbkey=hsa&keywords=GALNT8&mode=bfind)//[GALNTL1](http://www.genome.jp/dbget-bin/www_bfind_sub?mode=bfind&max_hit=10000&dbkey=hsa&keywords=GALNTL1&mode=bfind)//[GCNT1](http://www.genome.jp/dbget-bin/www_bfind_sub?mode=bfind&max_hit=10000&dbkey=hsa&keywords=GCNT1&mode=bfind) |

#### Mimic-vs-Control (up)

| **PathwayID** | **Definition** | **Fisher-Pvalue** | **SelectionCounts** | **SelectionSize** | **Count** | **Size** | **FDR** | **Enrichment_Score** | **Genes** |
| --- | --- | --- | --- | --- | --- | --- | --- | --- | --- |
| [hsa04722](http://www.genome.jp/dbget-bin/show_pathway?hsa04722+818+4214+4217+9261+23533+10603) | Neurotrophin signaling pathway - Homo sapiens (human) | 3.002889e-002 | 6 | 115 | 127 | 6205 | 1.000000e+000 | 1.522461e+000 | [CAMK2G](http://www.genome.jp/dbget-bin/www_bfind_sub?mode=bfind&max_hit=10000&dbkey=hsa&keywords=CAMK2G&mode=bfind)//[MAP3K1](http://www.genome.jp/dbget-bin/www_bfind_sub?mode=bfind&max_hit=10000&dbkey=hsa&keywords=MAP3K1&mode=bfind)//[MAP3K5](http://www.genome.jp/dbget-bin/www_bfind_sub?mode=bfind&max_hit=10000&dbkey=hsa&keywords=MAP3K5&mode=bfind)//[MAPKAPK2](http://www.genome.jp/dbget-bin/www_bfind_sub?mode=bfind&max_hit=10000&dbkey=hsa&keywords=MAPKAPK2&mode=bfind)//[PIK3R5](http://www.genome.jp/dbget-bin/www_bfind_sub?mode=bfind&max_hit=10000&dbkey=hsa&keywords=PIK3R5&mode=bfind)//[SH2B2](http://www.genome.jp/dbget-bin/www_bfind_sub?mode=bfind&max_hit=10000&dbkey=hsa&keywords=SH2B2&mode=bfind) |
| [hsa04614](http://www.genome.jp/dbget-bin/show_pathway?hsa04614+4142+5972) | Renin-angiotensin system - Homo sapiens (human) | 3.863077e-002 | 2 | 115 | 17 | 6205 | 1.000000e+000 | 1.413067e+000 | [MAS1](http://www.genome.jp/dbget-bin/www_bfind_sub?mode=bfind&max_hit=10000&dbkey=hsa&keywords=MAS1&mode=bfind)//[REN](http://www.genome.jp/dbget-bin/www_bfind_sub?mode=bfind&max_hit=10000&dbkey=hsa&keywords=REN&mode=bfind) |
| [hsa03320](http://www.genome.jp/dbget-bin/show_pathway?hsa03320+1579+2170+5346+5467) | PPAR signaling pathway - Homo sapiens (human) | 4.211539e-002 | 4 | 115 | 71 | 6205 | 1.000000e+000 | 1.375559e+000 | [CYP4A11](http://www.genome.jp/dbget-bin/www_bfind_sub?mode=bfind&max_hit=10000&dbkey=hsa&keywords=CYP4A11&mode=bfind)//[FABP3](http://www.genome.jp/dbget-bin/www_bfind_sub?mode=bfind&max_hit=10000&dbkey=hsa&keywords=FABP3&mode=bfind)//[PLIN1](http://www.genome.jp/dbget-bin/www_bfind_sub?mode=bfind&max_hit=10000&dbkey=hsa&keywords=PLIN1&mode=bfind)//[PPARD](http://www.genome.jp/dbget-bin/www_bfind_sub?mode=bfind&max_hit=10000&dbkey=hsa&keywords=PPARD&mode=bfind) |
| [hsa04810](http://www.genome.jp/dbget-bin/show_pathway?hsa04810+9564+1793+2253+3679+3683+5062+56924+23533) | Regulation of actin cytoskeleton - Homo sapiens (human) | 4.246657e-002 | 8 | 115 | 212 | 6205 | 1.000000e+000 | 1.371953e+000 | [BCAR1](http://www.genome.jp/dbget-bin/www_bfind_sub?mode=bfind&max_hit=10000&dbkey=hsa&keywords=BCAR1&mode=bfind)//[DOCK1](http://www.genome.jp/dbget-bin/www_bfind_sub?mode=bfind&max_hit=10000&dbkey=hsa&keywords=DOCK1&mode=bfind)//[FGF8](http://www.genome.jp/dbget-bin/www_bfind_sub?mode=bfind&max_hit=10000&dbkey=hsa&keywords=FGF8&mode=bfind)//[ITGA7](http://www.genome.jp/dbget-bin/www_bfind_sub?mode=bfind&max_hit=10000&dbkey=hsa&keywords=ITGA7&mode=bfind)//[ITGAL](http://www.genome.jp/dbget-bin/www_bfind_sub?mode=bfind&max_hit=10000&dbkey=hsa&keywords=ITGAL&mode=bfind)//[PAK2](http://www.genome.jp/dbget-bin/www_bfind_sub?mode=bfind&max_hit=10000&dbkey=hsa&keywords=PAK2&mode=bfind)//[PAK6](http://www.genome.jp/dbget-bin/www_bfind_sub?mode=bfind&max_hit=10000&dbkey=hsa&keywords=PAK6&mode=bfind)//[PIK3R5](http://www.genome.jp/dbget-bin/www_bfind_sub?mode=bfind&max_hit=10000&dbkey=hsa&keywords=PIK3R5&mode=bfind) |
